# Border-zone cardiomyocytes and macrophages regulate extracellular matrix remodeling to promote cardiomyocyte protrusion during cardiac regeneration

**DOI:** 10.1038/s41467-025-59169-4

**Published:** 2025-04-23

**Authors:** Florian Constanty, Bailin Wu, Ke-Hsuan Wei, I-Ting Lin, Julia Dallmann, Stefan Guenther, Till Lautenschlaeger, Rashmi Priya, Shih-Lei Lai, Didier Y. R. Stainier, Arica Beisaw

**Affiliations:** 1 Mechanisms of Cardiac Regeneration and Repair Lab, Institute of Experimental Cardiology, Heidelberg University, Heidelberg, Germany; 2 Helmholtz-Institute for Translational AngioCardioScience (HI-TAC) of the Max Delbrück Center for Molecular Medicine in the Helmholtz Association (MDC) at Heidelberg University, Heidelberg, Germany; 3 DZHK (German Centre for Cardiovascular Research), Partner site Heidelberg/Mannheim, Heidelberg, Germany; 4 Institute of Biomedical Sciences, Academia Sinica, Taipei, Taiwan; 5 Department of Developmental Genetics, Max Planck Institute for Heart and Lung Research, Bad Nauheim, Germany; 6 Deep Sequencing Platform, Max Planck Institute for Heart and Lung Research, Bad Nauheim, Germany; 7 DZHK (German Centre for Cardiovascular Research), Partner site Rhein/Main, Rhein/Main, Germany; 8 Cardio-Pulmonary Institute, Bad Nauheim, Germany; 9 Present Address: The Francis Crick Institute, London, UK

**Keywords:** Extracellular matrix, Cardiac regeneration, Monocytes and macrophages

## Abstract

Despite numerous advances in our understanding of zebrafish cardiac regeneration, an aspect that remains less studied is how regenerating cardiomyocytes invade and replace the collagen-containing injured tissue. Here, we provide an in-depth analysis of the process of cardiomyocyte invasion. We observe close interactions between protruding border-zone cardiomyocytes and macrophages, and show that macrophages are essential for extracellular matrix remodeling at the wound border zone and cardiomyocyte protrusion into the injured area. Single-cell RNA-sequencing reveals the expression of *mmp14b*, encoding a membrane-anchored matrix metalloproteinase, in several cell types at the border zone. Genetic *mmp14b* mutation leads to decreased macrophage recruitment, collagen degradation, and subsequent cardiomyocyte protrusion into injured tissue. Furthermore, cardiomyocyte-specific overexpression of *mmp14b* is sufficient to enhance cardiomyocyte invasion into the injured tissue and along the apical surface of the wound. Altogether, our data provide important insights into the mechanisms underlying cardiomyocyte invasion of the collagen-containing injured tissue during cardiac regeneration.

## Abstract (web summary)

Adult zebrafish are capable of heart regeneration, but how the collagenous injury site is replaced remains unclear. Here they provide an in-depth analysis of cardiomyocyte protrusion during zebrafish cardiac regeneration and highlight the role of macrophages and Mmp14b in ECM remodeling to support replacement of injured tissue with new cardiomyocytes.

## Introduction

Despite numerous advances in our understanding of cardiovascular biology, heart disease remains the leading cause of morbidity and mortality worldwide^1,2^. Following acute myocardial infarction in the human heart, millions of cardiomyocytes are lost and replaced with a fibrotic scar. While this fibrotic scar is necessary to maintain the structural integrity of the heart, its presence, in combination with decreased contractile mass, eventually leads to heart failure. There remains a pressing need to develop therapeutic strategies to replace lost heart tissue. Non-mammalian vertebrate organisms, such as the teleost zebrafish, have the ability to regenerate lost cardiomyocytes following multiple types of injury^3,4^. Notably, zebrafish can regenerate their hearts following cryoinjury, an injury model whereby a liquid nitrogen-cooled probe placed on the heart leads to cell death, activation of an inflammatory response, and deposition of a fibrotic collagen-containing scar, similar to myocardial infarction in the human heart^5–7^. While research efforts in the last two decades have led to an immense increase in our knowledge of the mechanisms of cardiac regeneration^8^, it remains unclear how regenerating cardiomyocytes at the wound border invade and eventually replace the collagen-containing scar tissue following injury.

Previous studies in zebrafish using photo-convertible Kaede protein have reported that zebrafish cardiomyocytes on the surface of the heart migrate, or are displaced, from the wound border to the injured area during the regeneration process^9^. This migration was shown to rely on the chemokine receptor Cxcr4, as treatment of adult fish with a CXCR4 antagonist following cardiac injury abrogated the presence of photo-converted cardiomyocyte-specific Kaede in the injured area. Furthermore, treatment with a CXCR4 antagonist led to a defect in scar resolution without affecting cardiomyocyte proliferation^9^. In neonatal mouse hearts, which can also regenerate following resection of the apex, cardiomyocytes protruding into the wound were shown to be essential for cardiac regeneration^10^. These protrusions were shown to rely on the dystrophin glycoprotein complex that links the actin cytoskeleton to the extracellular matrix (ECM), as mutant cardiomyocytes harboring a null *Dystrophin* mutation (from *mdx* mutant mice) lacked cardiomyocyte protrusions and isolated *mdx* mutant cardiomyocytes were not able to migrate into a collagen gel. Despite normal cardiomyocyte proliferation, *mdx* mutant mice retained a fibrotic scar^10^. These studies indicate that cardiomyocyte migration and invasion are necessary for resolution of the fibrotic scar during the cardiac regeneration process. Furthermore, results of these published studies suggest that cardiomyocyte proliferation alone is not sufficient to promote scar resolution following injury.

Notably, in two genetic mouse models that can promote regeneration in the nonregenerative adult mouse heart, cardiomyocyte protrusion and migration were also observed: first, activation of the transcription factor YAP in cardiomyocytes from YAP5SA overactivation or *Hippo*-deficient genetic models leads to cardiomyocyte proliferation and regeneration in the adult mouse heart^11–13^. YAP binds to several target genes to activate their expression in cardiomyocytes following myocardial infarction, including those that encode regulators of cytoskeletal dynamics^10^. *Hippo*-deficient cardiomyocytes extend protrusions into the infarcted area and isolated *Hippo-*deficient cardiomyocytes are able to migrate through a collagen gel matrix in vitro^10^. In another genetic model, cardiomyocyte-specific expression of a constitutively active form of *Erbb2* (caERBB2) is able to promote cardiomyocyte regeneration in the adult mouse heart^14^. Mechanistic analyses revealed that caERBB2 drives the activation of YAP, promotes the migration of isolated cardiomyocytes in vitro, and upregulates the expression of several genes that encode regulators of the extracellular matrix, including *Loxl2, Mmp14, Mmp2*, and *Pcolce*^15^. These studies suggest that cardiomyocyte protrusion and migration are regulated by genetic programs that can promote cardiac regeneration in the adult mammalian heart.

Following cryoinjury in the zebrafish heart, multiple cell types orchestrate the response to injury^8^. For example, endothelial, endocardial, and epicardial cells have been shown to provide growth factors and signaling molecules to promote revascularization of the injured tissue and cardiomyocyte regeneration^16–21^. Fibroblasts, which largely arise from the epicardium^22,23^, have been shown to deposit ECM to support the heart during the process of cardiac regeneration, and are essential for cardiomyocyte proliferation following injury^23–26^. The importance of immune cells in promoting the cardiac regeneration process has also been recognized in more recent years. In particular, macrophages have been shown to play an essential role in regeneration of zebrafish, salamander, and neonatal mouse hearts in response to injury^27–30^. Macrophages have been shown to directly contribute to and regulate the composition of scar/ECM in the cryoinjured heart^28,31,32^. Furthermore, depletion of macrophages by clodronate liposome administration or the use of genetic models leads to defects in CM proliferation and neovascularization in regenerating hearts^27,29,33,34^. While it is clear that many, if not all, cardiac cell types play an essential role in the process of cardiac regeneration, their contribution to cardiomyocyte repopulation of fibrotic tissue following cryoinjury is largely unknown.

Here, we provide an in-depth analysis of cardiomyocyte protrusion into the injured tissue during zebrafish cardiac regeneration. We show that cardiomyocytes at the wound border exhibit characteristics of migratory cells, including motile filopodia-like extensions into the injured tissue and upregulation of gene expression programs regulating actin dynamics, focal adhesions, and ECM remodeling. Furthermore, we show that macrophages play an essential role at the border zone to remodel the ECM and promote cardiomyocyte protrusion. We then show that Mmp14b is an important regulator of macrophage presence and ECM remodeling at the border zone, and subsequent CM invasion into the injured tissue during cardiac regeneration in zebrafish.

## Results

### Characterization of CM protrusion into injured tissue

**Fig. 1:**
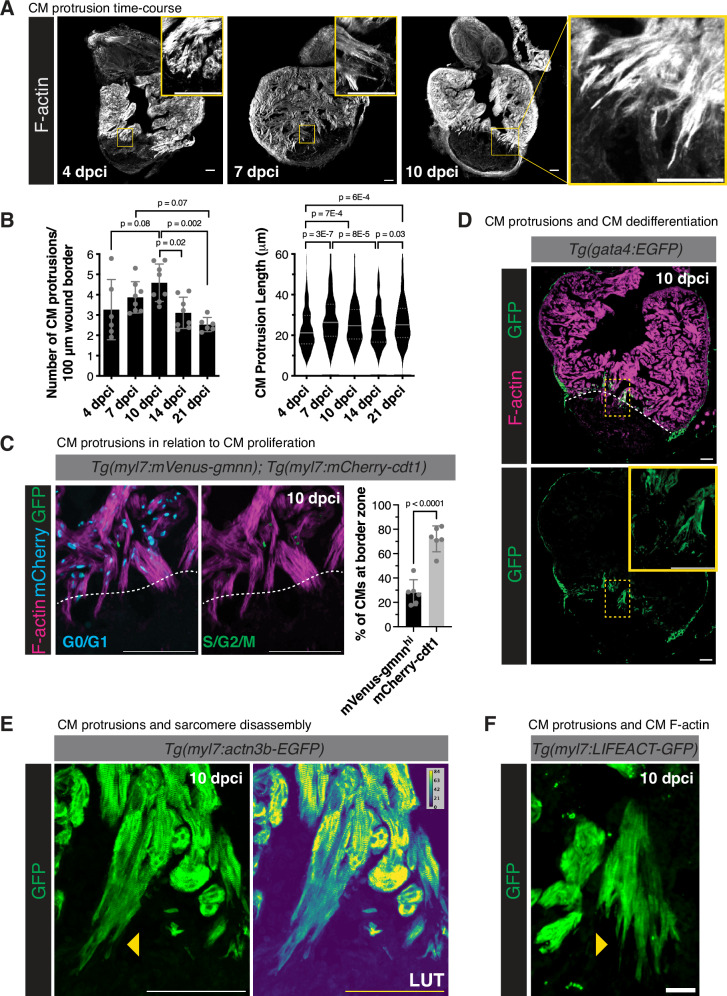
Characterization of CM protrusion into the injured area during zebrafish heart regeneration. **A** Phalloidin staining of F-actin in thick cryosections of zebrafish ventricles at 4, 7, and 10 days post cryoinjury (dpci). Yellow box denotes the zoomed image from the wound border zone. **B** Quantification of the number of CM protrusions per 100 micron of wound border (left) and length of CM protrusions (right) from thick cryosections of adult ventricles at 4 (*n* = 6 ventricles), 7 (*n* = 8 ventricles), 10 (*n* = 8 ventricles), 14 (*n* = 8 ventricles), and 21 (*n* = 6 ventricles) dpci. Length of CM protrusions were quantified at 4 (*n* = 245 CM protrusions from 6 ventricles), 7 (*n* = 323 CM protrusions from 8 ventricles), 10 (*n* = 449 CM protrusions from 8 ventricles), 14 (*n* = 300 CM protrusions from 8 ventricles), and 21 (*n* = 248 CM protrusions from 6 ventricles) dpci. Data are presented as mean ± SD (number of CM protrusions) and violin plots of all points with solid gray lines indicating the median and dotted gray lines indicating 25th and 75th percentile (length of CM protrusions). *P*-values were calculated using one-way ANOVA with Tukey’s multiple comparisons test (number of CM protrusions) and the Kruskal–Wallis test with Dunnett’s multiple comparisons test (CM protrusion length). Source data are presented in the Source Data file. **C** Immunostaining of GFP, mCherry, and F-actin in *Tg(myl7:mVenus-gmnn); Tg(myl7:mCherry-cdt1)* ventricles at 10 dpci. White dashed line indicates the injury border. The percentages of mVenus-Gmnn^hi^ and mCherry-Cdt1+ CM nuclei directly neighboring the wound border (*n* = 6 ventricles) were quantified on the right. Data are presented as mean ± SD. *P*-value was calculated using an unpaired two-sided *t*-test. Source data are presented in the Source Data file. **D** GFP and Phalloidin staining of *Tg(gata4:EGFP)* ventricles at 10 dpci. White dashed line indicates the injury border and the yellow box denotes the zoomed image from the wound border zone. **E** GFP staining in *Tg(myl7:actn3b-EGFP)* ventricles at 10 dpci marking the CM sarcomere. LUT (look-up table) images depict GFP intensity color-coded according to the scale within the image. Yellow arrowheads point to CM protrusions devoid of organized sarcomere structures. **F** GFP staining of *Tg(myl7:LIFEACT-GFP)* ventricles at 10 dpci marking CM-specific F-actin. Yellow arrowhead points to actin-filled CM protrusions. Scale bars: 100 μm in (**A**, **C**, and **D**), 20 μm in (**E** and **F**).

While previous studies have indicated that cardiomyocyte (CM) protrusion and invasion into the injured tissue are likely essential for cardiac regeneration^9,10,15^, we lack a detailed knowledge of the molecular mechanisms underlying these processes. To characterize CM protrusion, we performed a time-course analysis at multiple timepoints following cryoinjury. Using phalloidin, which labels actin filaments (F-actin) and is highly abundant in zebrafish cardiomyocytes (Supplementary Fig. 1A, B), we observed a peak in the number of CM protrusions at the wound border zone between 7 and 10 days post cryoinjury (dpci, Fig. 1A, B, and Supplementary Fig. 1C). Furthermore, we observed a peak in CM protrusion length at 7 dpci (Fig. 1B), corresponding to timepoints post cryoinjury that exhibit high levels of CM proliferation. To determine the relationship between protruding and proliferating CMs, we utilized the FUCCI (fluorescent ubiquitination-based cell cycle indicator) cell-cycle reporter line *Tg(myl7:mVenus-gmnn); Tg(myl7:mCherry-cdt1)*, marking CMs in S/G2/M phase with mVenus and CMs in G0/G1 phase with mCherry. Immunostaining of ventricular sections at 10 dpci revealed that approximately 70% of CM nuclei adjacent to CM protrusions into the injured area are in G0/G1 phase (Fig. 1C). These observations indicate that CM protrusion and proliferation are regenerative processes that are spatially uncoupled, and suggest that CM proliferation may precede protrusion into the injured tissue. Additional processes that have been described during cardiac regeneration include cardiomyocyte dedifferentiation and sarcomere disassembly^35–38^. We examined *Tg(gata4:EGFP)* ventricular sections at 10 dpci and found that nearly all CMs protruding into the injured area exhibited *gata4*:EGFP expression, indicating that CMs that invade the injured area have undergone dedifferentiation (Fig. 1D). Colocalization of protruding CMs with additional markers of dedifferentiation, including embryonic cardiac myosin heavy chain (embCMHC, N2.261)^39^, *mustn1b:*EGFP^40,41^, and the glycolytic marker *pkma*^41,42^ confirmed this result (Supplementary Fig. 1D, E). Further, we examined CM sarcomere structure using ventricular sections from *Tg(myl7:actn3b-EGFP)* zebrafish at 10 dpci and observed reduced Actn3b-EGFP intensity and a less regular banding pattern at the distal tips of CMs protruding into the injured tissue (Fig. 1E), indicative of sarcomere disassembly in line with our previously published data^43^. Lastly, we examined ventricular sections from *Tg(myl7:LIFEACT-GFP)* zebrafish at 10 dpci and found that invading CMs at the border zone extend actin-filled protrusions into the injured area (Fig. 1F).

**Fig. 2:**
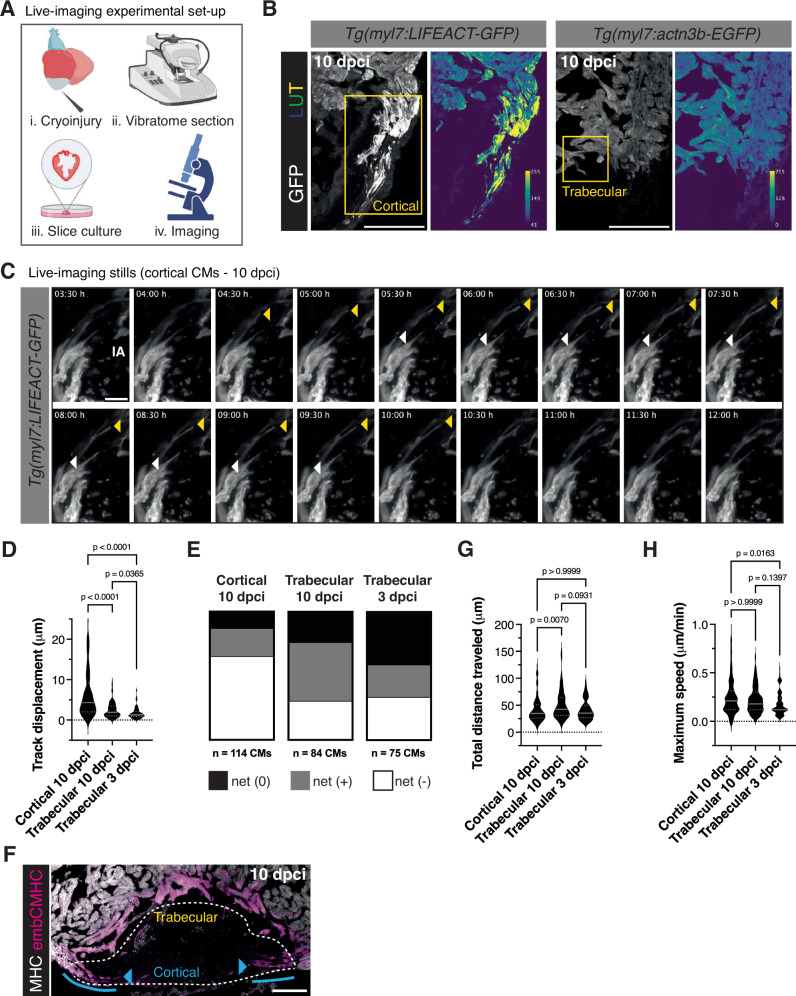
Live-imaging of CM protrusion into the injured tissue during zebrafish heart regeneration. **A** Schematic of the experiment illustrating vibratome sectioning of cryoinjured hearts, slice culture, and live imaging. Created in BioRender. Beisaw, A. (2025) https://BioRender.com/ypwjv2r. **B** GFP staining in thin sections of *Tg(myl7:LIFEACT-GFP)* (left) and *Tg(myl7:actn3b-EGFP)* (right) ventricles at 10 dpci. LUT (look-up table) images depict GFP intensity color-coded according to the scale within the image. The LUT is linear and covers the full range of the data. Yellow boxes denote the cortical/trabecular CM focus area of time-lapse imaging. **C** Time-lapse imaging of *Tg(myl7:LIFEACT-GFP)*+ cortical CMs at 10 dpci. Yellow arrowhead follows a CM protrusion that displays a net positive migration into the injured area and the white arrowhead follows a CM protrusion that displays a net negative migration away from the injured area (IA). **D** Quantification of track displacement from CM protrusions at the border zone in *Tg(myl7:LIFEACT-GFP)*+ cortical CMs at 10 dpci (*n* = 114 protrusions from 6 ventricles) and *Tg(myl7:actn3b-EGFP)*+ trabecular CMs at 3 (*n* = 76 protrusions from 5 ventricles) and 10 (*n* = 84 protrusions from 4 ventricles) dpci. Data are presented as violin plots of all points with solid gray lines indicating the median and dotted gray lines indicating 25th and 75th percentile. *P*-values were calculated using a Kruskal–Wallis test with Dunnett’s multiple comparisons test. Source data are presented in the Source Data file. **E** Distribution of tracked CM protrusions with a net positive displacement (> 1 μm into the injured area), net negative displacement (> 1 μm away from the injured area), or net zero displacement (<1 μm) comparing *t* = 0 and *t* = 12 h of live-imaging in *Tg(myl7:LIFEACT-GFP)*+ cortical CMs at 10 dpci (*n* = 114 protrusions from 6 ventricles) and *Tg(myl7:actn3b-EGFP)*+ trabecular CMs at 3 (*n* = 76 protrusions from 5 ventricles) and 10 (*n* = 84 protrusions from 4 ventricles) dpci. Source data are presented in the Source Data file. **F** Immunostaining of myosin heavy chain (MHC) and embryonic CMHC (embCMHC) in thin sections of a ventricle at 10 dpci. The white dotted line denotes the outline of the wound. Blue arrowheads point to cortically-located CMs and the blue line outlines the increased invasion of cortically-located CMs into the injured tissue. **G** Quantification of total distance traveled by tracked CM protrusions in *Tg(myl7:LIFEACT-GFP)*+ cortical CMs at 10 dpci (*n* = 114 protrusions from 6 ventricles) and *Tg(myl7:actn3b-EGFP)*+ trabecular CMs at 3 (*n* = 76 protrusions from 5 ventricles) and 10 (*n* = 84 protrusions from 4 ventricles) dpci. Data are presented as violin plots of all points with solid gray lines indicating the median and dotted gray lines indicating 25th and 75th percentile. *P*-values were calculated using a Kruskal–Wallis test with Dunnett’s multiple comparisons test. Source data are presented in the Source Data file. **H** Maximum speed measured in tracked CM protrusions *Tg(myl7:LIFEACT-GFP)*+ cortical CMs at 10 dpci (*n* = 114 protrusions from 6 ventricles) and *Tg(myl7:actn3b-EGFP)*+ trabecular CMs at 3 (*n* = 76 protrusions from 5 ventricles) and 10 (*n* = 84 protrusions from 4 ventricles) dpci. Data are presented as violin plots of all points with solid gray lines indicating the median and dotted gray lines indicating 25th and 75th percentile. *P*-values were calculated using a Kruskal–Wallis test with Dunnett’s multiple comparisons test. Source data are presented in the Source Data file. Scale bars: 100 μm in (**B** and **F**), 20 μm in (**C**).

To visualize the process of CM invasion in situ, we optimized a previously published protocol to image living ventricular slices from regenerating zebrafish hearts (Fig. 2A)^38^. First, we observed that *Tg(myl7:*LIFEACT-GFP*)* levels were particularly high in cortically-located CMs at the border zone (Fig. 2B). We subjected ventricular slices from *Tg(myl7:LIFEACT-GFP)* zebrafish at 10 dpci to live imaging and found that cortically-located CMs extended/retracted filopodia-like structures into and away from the injured collagenous tissue (Fig. 2C and Supplementary Videos 1 and 2). We manually tracked the ends of CM protrusions at the border zone over the entire 12-h time-lapse imaging period and found that the mean of cortically-located CM protrusion displacement (distance between the start and end point of protrusion at the end of the time-lapse period) was 6.447 μm, with a maximum displacement of 26.17 μm (Fig. 2D, Supplementary Fig. 2C and Supplementary Video 2). The majority of this displacement (65%) was in a net (−) direction, or away from the injured area when measuring the position of the protrusion end from the beginning to end of the time-lapse image (compared to 22% of displacement in a net (+) direction, or into the injured area, Fig. 2E). Movement of CM protrusions in a direction away from the injured tissue may reflect the previously described role for filopodia in environment sensing^44^; however, the 22% of CM protrusion movement into the injured tissue appears sufficient to promote CM invasion, as we often observe that cortically-located CMs exhibit high levels of invasion into the injured tissue in fixed ventricular sections at 10 dpci (Fig. 2F). In order to follow trabecular CM protrusion over time, we used living ventricular slices from *Tg(myl7:actn3b-EGFP)* zebrafish at 10 dpci, as we found that CM Actn3b-EGFP levels were higher in trabecular CMs at the border zone (Fig. 2B). Trabecular CMs at 10 dpci extended/retracted filopodia-like structures into and away from the injured collagenous tissue similar to cortically-located CMs (Supplementary Fig. 2A and Supplementary Video 3), but with a mean of protrusion displacement of 2.418 μm, and a maximum displacement of 9.063 μm (Fig. 2D). However, the majority of trabecular CM protrusion displacement was in a net (+) direction (46% compared to 30% in a net (−) direction) (Fig. 2E). We further tracked trabecular CM protrusion at 3 dpci (Supplementary Fig. 2B and Supplementary Video 4) and found that the mean of protrusion displacement was lower than that of 10 dpci (1.641 μm) and that 41% of protrusions had a net displacement less than 1 μm (net (0)) (Fig. 2E). Notably, the total distance traveled by cortically-located and trabecular CM protrusions did not correlate with track displacement, i.e., trabecular CMs at 10 dpci traveled the most distance over the measured time-course (mean = 49.89 μm, compared to mean = 40.82 μm in cortically-located CM protrusions and mean = 41.15 μm in trabecular CM protrusions at 3 dpci) despite having less track displacement (Fig. 2G). In line with these measurements, the confinement ratio (net distance/total distance traveled) was highest in cortically-located CM protrusions, suggesting that cortical CM protrusions were most efficient in being displaced from their initial location (Supplementary Fig. 2D). Lastly, we measured the maximum speed of CM protrusion movement and found that cortically-located CM protrusions moved at a maximum rate of 0.26 μm/min and trabecular CM protrusions with a maximum rate of 0.22 μm/min (Fig. 2H). Trabecular CM protrusions at 3 dpci moved more slowly, at a maximum rate of 0.18 μm/min (Fig. 2H). Altogether, these observations suggest that CMs at the border zone extend motile protrusions directed into the injured area that are reminiscent of filopodia on the leading edge of migrating cells.

### Macrophages are associated with CM protrusions

**Fig. 3:**
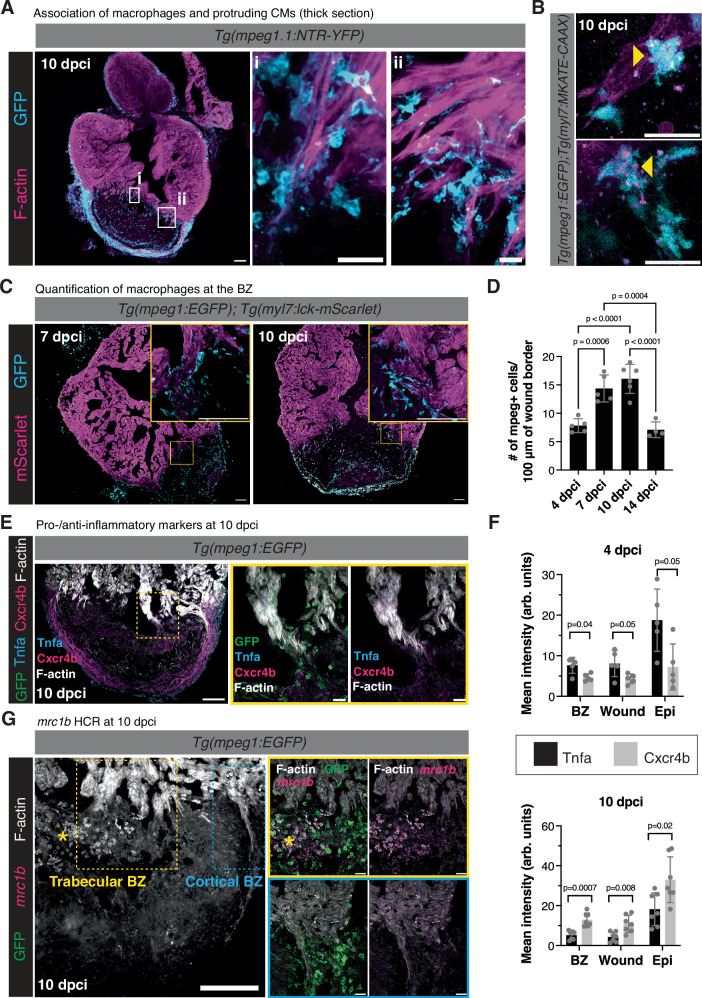
Macrophages are closely associated with protruding CMs at the border zone. **A** Immunostaining of GFP and F-actin in thick sections from *Tg(mpeg1.1:NTR-YFP)* ventricles at 10 dpci. White boxes denote the zoomed image from the wound border zone in (i) and (ii). **B** Still image from time-lapse imaging of *Tg(mpeg1:EGFP); Tg(myl7:mKATE-CAAX)* ventricular sections at 10 dpci. Yellow arrowheads point to *mpeg1*:EGFP+ cells containing *myl7*:mKATE-CAAX fluorescence. **C** Immunostaining of GFP and mScarlet in *Tg(mpeg1:EGFP); Tg(myl7:lck-mScarlet)* ventricles at 7 and 10 dpci. Yellow boxes denote the zoomed image from the wound border zone. **D** Quantification of the number of *mpeg1*:EGFP+ cells 50 μm proximal and distal to the wound border at 4 (*n* = 5 ventricles), 7 (*n* = 5 ventricles), 10 (*n* = 6 ventricles), and 14 dpci (*n* = 4 ventricles). Data are presented as mean ± SD. *P*-values were calculated using one-way ANOVA with Tukey’s multiple comparisons test. Source data are presented in the Source Data file. **E** Immunostaining of GFP, Tnfa, Cxcr4b, and F-actin in *Tg(mpeg1:EGFP)* ventricles at 10 dpci. Yellow box denotes the zoomed image from the wound border zone. **F** Quantification of mean Tnfa and Cxcr4b intensity (arbitrary units, arb. units) 50 μm proximal and distal to the wound border at 4 (*n* = 5 ventricles) and 10 (*n* = 7 ventricles) dpci. Data are presented as mean ± SD. *P*-values were calculated with unpaired two-sided *t*-tests. Source data are presented in the Source Data file. **G** *mrc1b* in situ hybridization chain reaction (HCR) and immunostaining for GFP and F-actin in *Tg(mpeg1:EGFP)* ventricles at 10 dpci. The yellow dotted box denotes the zoomed image from the trabecular BZ and the blue dotted box denotes the zoomed image from the cortical BZ. *denotes background autofluorescence from erythrocytes. Scale bars: 100 μm in (**A**, **C**, **E**), and **G**), 20 μm in **(Ai)** and **(Aii)**, (**B**), and zoomed images in (**E** and **G**).

In the course of the live-imaging analysis above, we observed phagocytosed CM material in cells next to protruding cardiomyocytes. Using transgenic lines, marking CM membrane and macrophages, we found that macrophages are in very close proximity to protruding CMs at the wound border zone (Fig. 3A). We also observed macrophages with CM membrane material in their cell body in time-lapse imaging from protruding CMs, further suggesting that these cell types are in very close proximity (Fig. 3B and Supplementary Video 5). Quantification of macrophages 50 μm proximal and distal to the wound border revealed a peak in macrophage number at 7 and 10 dpci, corresponding to the peak in CM protrusion (Fig. 3C, D). To further understand the interaction of CMs and macrophages at the border zone, we performed time-lapse imaging of living ventricular sections from *Tg(mpeg1:EGFP); Tg(myl7:lck-mScarlet)* hearts at 10 dpci. Macrophages at the border zone extended multiple filopodia and were in close contact to CMs at the border zone (Supplementary Fig. 3A and Supplementary Video 6). There was a striking difference in macrophage morphology in macrophages contacting CMs at the border zone and macrophages within the injured area (Supplementary Fig. 3A). Macrophages within the injured area were significantly rounder (Supplementary Fig. 3B and Supplementary Video 7) and less protrusive. Previous studies have shown a correlation between macrophage morphology and phenotypic state, with anti-inflammatory or pro-healing macrophages displaying a more elongated morphology^45^. Immunostaining of the pro-inflammatory cytokine Tnfa and the anti-inflammatory macrophage marker Cxcr4b revealed an enrichment of Cxcr4b localization at the wound border zone at 10 dpci (Fig. 3E). Quantification of Tnfa intensity revealed that at 4 dpci, a timepoint where fewer CM protrusions are observed, there was an enrichment of pro-inflammatory Tnfa intensity at the border zone, within the wound, and along the epicardial surface of the heart. Conversely, at 10 dpci, when CM protrusion number and length peak, we observed an enrichment of Cxcr4b intensity at the border zone, within the wound, and along the epicardial surface of the heart compared to Tnfa (Fig. 3F and Supplementary Fig. 3C, D). Notably, measurement of macrophage aspect ratio did not reveal a difference in roundness of macrophages at the border zone at 4 versus 10 dpci, despite the difference in pro- and anti-inflammatory macrophage marker localization (Supplementary Fig. 3E). These results suggest that unlike in other model systems, macrophage aspect ratio does not correspond to pro-/anti-inflammatory marker localization in the regenerating zebrafish heart. We further utilized in situ hybridization chain reaction (HCR) using a probe against *mrc1b*, an alternatively activated macrophage marker associated with phagocytic macrophages^46^, and found that *mrc1b*+ macrophages were present at the border zone and associated with protruding CMs at 10 dpci (Fig. 3G). Together, these data indicate a correlation between the presence of anti-inflammatory, or pro-healing, macrophages at the border zone at peak timepoints of CM protrusion into the injured area.

**Fig. 4:**
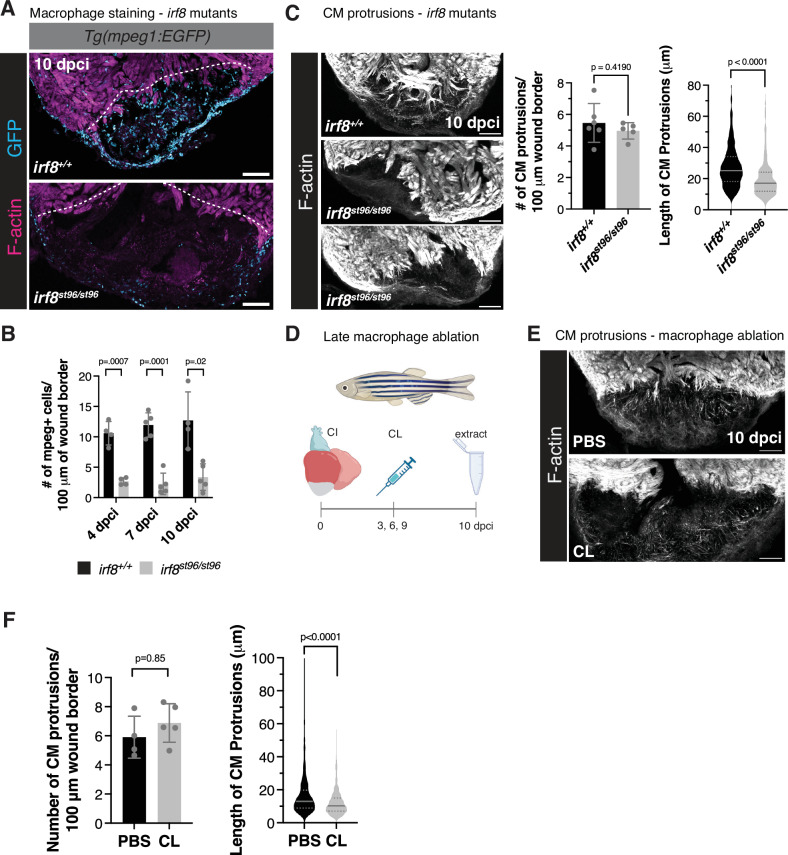
Macrophages are required for CM protrusion into the injured area. **A** Immunostaining of GFP and F-actin in *Tg(mpeg1:EGFP)* ventricles from *irf8*^*st96/st96*^ mutants and wild-type siblings at 10 dpci. White dotted line denotes the approximate wound border. **B** Quantification of *mpeg1*:EGFP+ cells 50 μm proximal and distal to the wound border in *Tg(mpeg1:EGFP)* ventricles from *irf8*^*st96/st96*^ mutants and wild-type siblings at 4 (*n* = 4 wild-type, *n* = 4 mutant ventricles), 7 (*n* = 5 wild-type, *n* = 5 mutant ventricles), and 10 (*n* = 4 wild-type, *n* = 5 mutant ventricles) dpci. Data are presented as mean ± SD. *P*-values were calculated using multiple unpaired two-sided *t*-tests and corrected for multiple comparisons using the Bonferroni-Dunn method. Source data are presented in the Source Data file. **C** Phalloidin staining of F-actin in thick cryosections of zebrafish ventricles from *irf8*^*st96/st96*^ mutants and wild-type siblings at 10 dpci (left). Quantification of the number of CM protrusions per 100 micron of wound border (middle) and length of CM protrusions (right) from thick cryosections of *irf8*^*st96/st96*^ mutant and wild-type sibling ventricles at 10 dpci (*n* = 485 CM protrusions from 6 wild-type ventricles, *n* = 240 CM protrusions from 5 mutant ventricles). Data are presented as mean ± SD (number of CM protrusions) and violin plots of all points with solid gray lines indicating the median and dotted gray lines indicating 25th and 75th percentile (length of CM protrusions). *P*-values were calculated using an unpaired two-sided *t*-test (number of CM protrusions) and a two-sided Mann–Whitney test (CM protrusion length). Source data are presented in the Source Data file. **D** Schematic illustrating the experimental set-up to ablate macrophages using clodronate liposomes (CL). Created in BioRender. Beisaw, A. (2025) https://BioRender.com/2iptntt. **E** Phalloidin staining of F-actin in thick cryosections of zebrafish ventricles treated with PBS liposomes or clodronate liposomes at 10 dpci. **F** Quantification of the number of CM protrusions per 100 micron of wound border (left) and length of CM protrusions (right) from thick cryosections of ventricles from PBS (*n* = 521 CM protrusions from 4 ventricles) and CL (*n* = 664 CM protrusions from 5 ventricles) treated fish at 10 dpci. Data are presented as mean ± SD (number of CM protrusions) and violin plots of all points with solid gray lines indicating the median and dotted gray lines indicating 25th and 75th percentile (CM protrusion length). *P*-values were calculated using an unpaired two-sided *t*-test (number of CM protrusions) and a two-sided Mann–Whitney test (CM protrusion length). Source data are presented in the Source Data file. PBS PBS liposomes, CL clodronate liposomes. Scale bars: 100 μm.

In order to determine whether macrophages contribute to the CM protrusion process, we utilized *irf8*^*st96/st96*^ mutant zebrafish^47^, which largely lack *mpeg1*+ macrophages in the heart at multiple timepoints following cryoinjury (Fig. 4A, B). Following cryoinjury in *irf8*^*st96/st96*^ mutant zebrafish, we observed that while the number of CM protrusions was not affected at 10 dpci, the length of CM protrusions was significantly decreased in the absence of macrophages when compared to *irf8*^*+/+*^ siblings (Fig. 4C). Because a lack of macrophages in the *irf8*^*st96/st96*^ mutant hearts may alter cardiac physiology/homeostasis and the inflammatory response to cardiac injury^29,48,49^, we confirmed our results in a *Tg(mpeg1.1:NTR-YFP)* transgenic line, harboring macrophage-specific expression of the bacterial nitroreductase (NTR) enzyme. Treatment of adult fish with nifurpirinol, a nitroaromatic compound that was shown to be superior to metronidazole for NTR-mediated cell ablation^50^, from 4 to 6 dpci resulted in a large variability in macrophage ablation efficiency. However, we observed a significant correlation in the number of macrophages present at the wound border and the length in CM protrusions and no correlation between macrophage presence and number of CM protrusions, similar to the *irf8*^*st96/st96*^ mutant (Supplementary Fig. 4A, B). We further performed intraperitoneal injections of adult zebrafish with clodronate liposomes (CL) to deplete macrophages at later stages of heart regeneration. While CL have been shown to affect neutrophils^51^, we administered CL starting at 3 dpci (Fig. 4D), a timepoint following the peak of neutrophil presence in the adult zebrafish heart^29,32^. In line with the results described above, we again observed no difference in CM protrusion number and a significant decrease in CM protrusion length in regenerating zebrafish hearts at 10 dpci (Fig. 4E, F). Altogether, these results indicate that the absence of macrophages in the regenerating zebrafish heart leads to defects in CM protrusion length.

### Resident macrophages regulate ECM remodeling and CM protrusion

**Fig. 5:**
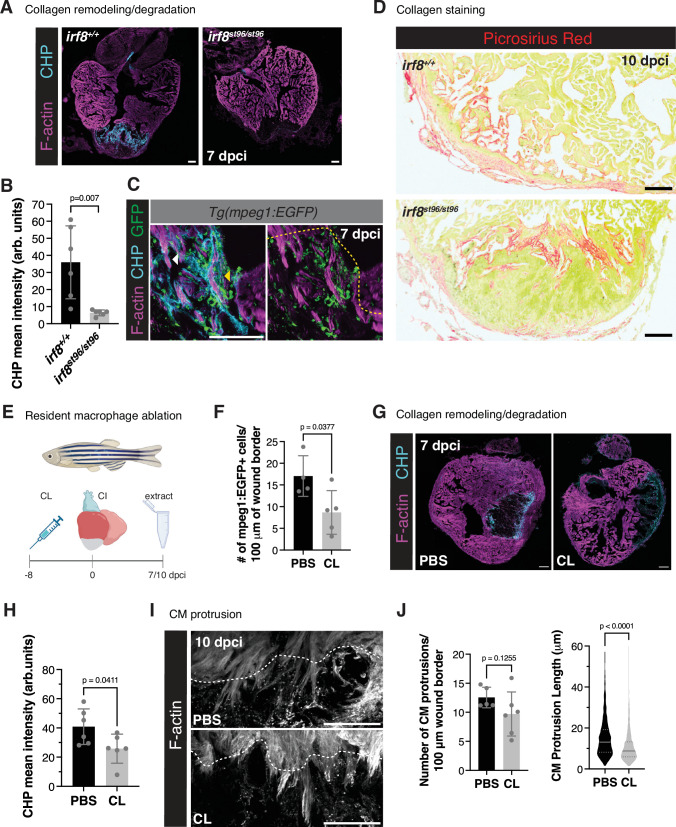
Macrophages are required for collagen remodeling at the wound border zone. **A** Collagen hybridizing peptide (CHP) and F-actin staining of *irf8*^*st96/st96*^ mutant and wild-type sibling ventricles at 7 dpci. **B** Quantification of CHP intensity (arbitrary units, arb. units) at the border zone of *irf8*^*st96/st96*^ mutant (*n* = 6 ventricles) and wild-type sibling (*n* = 6 ventricles) at 7 dpci. Data are presented as mean ± SD. *P*-values were calculated using an unpaired two-sided *t*-test. Source data are presented in the Source Data file. **C** Immunostaining of GFP, CHP, and F-actin in *Tg(mpeg1:EGFP)* ventricles at 7 dpci. Yellow dotted lines denotes the approximate injury plane at the border zone. Yellow arrowhead points to high CHP staining at areas of CM-macrophage interaction and white arrowhead points to CHP staining at the leading edge of a protruding CM without associated macrophages. **D** Picrosirius red staining of collagen in *irf8*^*st96/st96*^ mutant and wild-type sibling ventricles at 10 dpci. **E** Schematic illustrating the experimental scheme to deplete resident macrophages with clodronate (CL) or PBS control liposomes. Created in BioRender. Beisaw, A. (2025) https://BioRender.com/4qhphx7. **F** Quantification of the number of *mpeg1*:EGFP+ cells 50 μm proximal and distal to the wound border in ventricles at 7 dpci in CL- (*n* = 5 ventricles) and PBS-liposome (*n* = 4 ventricles) injected fish. Data are presented as mean ± SD. *P*-value was calculated using an unpaired two-sided *t*-test. Source data are presented in the Source Data file. **G** Collagen hybridizing peptide (CHP) and F-actin staining of PBS control and CL-injected ventricles at 7 dpci. **H** Quantification of CHP intensity (arbitrary units, arb. units) at the border zone of 7 dpci ventricles in CL- (*n* = 6 ventricles) and PBS-liposome (*n* = 6 ventricles) injected fish. Data are presented as mean ± SD. *P*-value was calculated using an unpaired two-sided *t*-test. Source data are presented in the Source Data file. **I** Phalloidin staining of F-actin in thick cryosections of PBS liposome-injected and CL-injected zebrafish ventricles at 10 dpci. White dashed lines indicate the injury border. **J** Quantification of the number of CM protrusions per 100 micron of wound border (left) and length of CM protrusions (right) from thick cryosections of PBS liposome-injected (*n* = 598 CM protrusions from 5 ventricles) and CL-injected (*n* = 845 CM protrusions from 6 ventricles) zebrafish at 10 dpci. Data are presented as mean ± SD (number of CM protrusions) and violin plots of all points with solid gray lines indicating the median and dotted gray lines indicating 25th and 75th percentile (CM protrusion length). *P*-values were calculated using an unpaired two-sided *t*-test (number of CM protrusions) and a two-sided Mann–Whitney test (CM protrusion length). Source data are presented in the Source Data file. Scale bars: 100 μm.

Based on our histological and live-imaging analyses, we hypothesized that the extracellular matrix (ECM) microenvironment adjacent to the border zone is important for CM protrusion/invasion and, further, that this ECM microenvironment is disrupted in the absence of macrophages. To address this hypothesis, we used a collagen hybridizing peptide (CHP) to visualize remodeling or degrading collagen at the border zone of regenerating hearts^52^. In *irf8*^*+/+*^ wild-type ventricles, we observed collagen degradation/remodeling at the wound border zone (associated with protruding CMs) and extending into the wound. Strikingly, in *irf8*^*st96/st96*^ mutant ventricles, there was a complete loss of CHP staining of collagen degradation/remodeling (Fig. 5A, B), suggesting that macrophage presence is necessary for ECM remodeling at the border zone and within the wound. To determine the relationship between protruding CMs, macrophages, and collagen remodeling/degradation at the border zone, we stained ventricles from *Tg(mpeg1:EGFP)* fish at 7 dpci and observed that while collagen degradation/remodeling is present at the leading edge of protruding CMs at regions without associated macrophages, the intensity of CHP signal is very high at regions where macrophages and protruding CMs interact (Fig. 5C). As macrophages have previously been shown to deposit collagen in the wound area following cryoinjury in zebrafish hearts^31^, we stained *irf8*^*+/+*^ wild-type and *irf8*^*st96/st96*^ mutant ventricles with Picrosirius red to determine collagen content in the regenerating heart. We observed that collagen was localized at the wound border of *irf8*^*st96/st96*^ mutant ventricles and at similar levels compared to *irf8*^*+/+*^ wild-type ventricles (Fig. 5D and Supplementary Fig. 4C), although the distribution of collagen differed slightly in the absence of macrophages. This difference in localization was likely due to the remaining presence of a large fibrin clot within the wound of *irf8*^*st96/st96*^ mutant ventricles (Supplementary Fig. 4D). Macrophage presence is essential for organismal survival following cryoinjury, as a large majority of *irf8*^*st96/st96*^ mutant fish died at mid-late stages of cardiac regeneration (Supplementary Fig. 4E). As macrophages have also been shown to play a role in the activation of cardiac fibroblasts^52,53^, we used quantitative reverse-transcription PCR (RT-qPCR) to analyze fibroblast gene expression in *irf8*^*+/+*^ wild-type compared to *irf8*^*st96/st96*^ mutant ventricles. We observed increased expression of canonical fibroblast markers, including *vim, acta2, col1a1a*, and *c4* in *irf8*^*st96/st96*^ mutant ventricles compared to wild-type siblings at 10 dpci (Supplementary Fig. 4F). Furthermore, we observed an increase in fibroblast genes that have been shown in previous studies to promote regeneration, including *fn1a, fn1b*, and *col12a1a* (Supplementary Fig. 4F), suggesting that the lack of macrophages in *irf8*^*st96/st96*^ mutants does not result in a decrease in fibroblast activation or expression of regenerative fibroblast marker genes. Altogether, these results indicate that macrophages are essential for collagen degradation/remodeling at the border zone, and that the lack of collagen degradation/remodeling in *irf8*^*st96/st96*^ mutant ventricles is not due to the absence of collagen in the wound or a decrease in fibroblast gene expression programs.

Recently published studies have shown that resident macrophages are essential for the cardiac regenerative response^30^. In order to determine whether resident macrophages are important for ECM remodeling and CM protrusion, we administered clodronate liposomes (CL) 8 days prior to cryoinjury to deplete resident cardiac macrophages, performed cryoinjury, and determined the effect on ECM remodeling and CM protrusion at 7 and 10 dpci (Fig. 5E). Quantification of *Tg(mpeg1:EGFP)*+ cells at the wound border zone revealed that depletion of the resident macrophage population with CL resulted in a decrease of macrophages at the border zone (Fig. 5F). Notably, staining of remodeling/degrading collagen with CHP revealed a significant decrease in CHP intensity at the border zone in resident macrophage-depleted hearts at 7 dpci (Fig. 5G, H). Accordingly, we observed no significant decrease in the number of CM protrusions, but a decrease in CM protrusion length at 10 dpci at the border zone (Fig. 5I, J), similar to *irf8*^*st96/st96*^ mutant ventricles that largely lack macrophages. Altogether, these data indicate that macrophage presence, and in particular resident macrophage presence, at the wound border zone is essential for remodeling the ECM and CM protrusion/invasion.

### Border zone-specific gene expression signatures

**Fig. 6:**
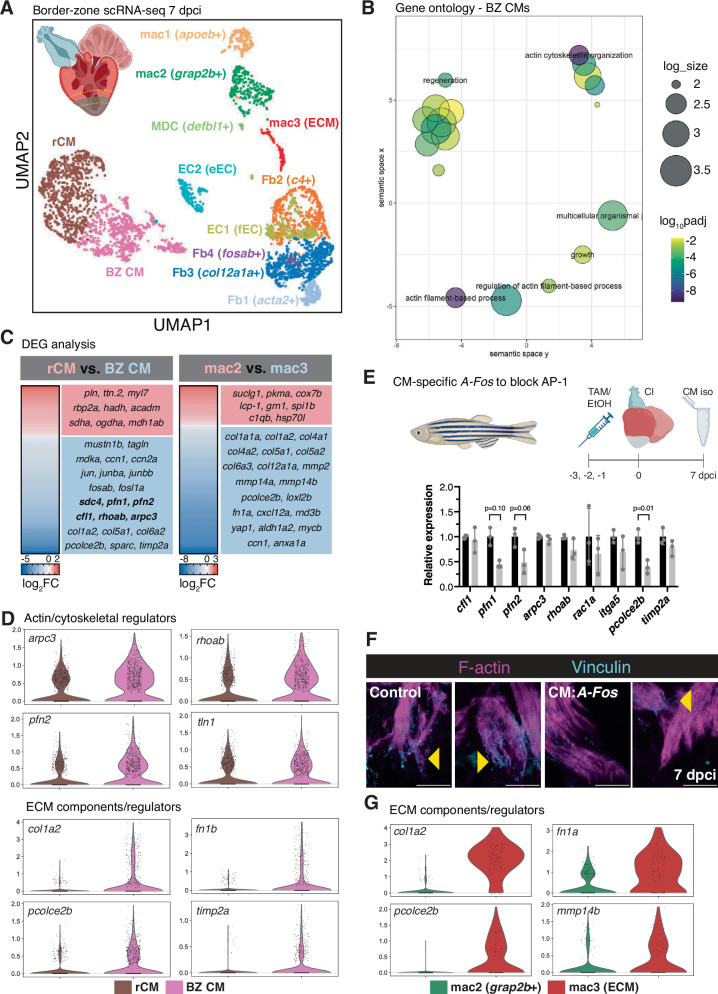
Single-cell RNA-sequencing reveals border zone-specific gene expression signatures in the regenerating heart. **A** UMAP plot of scRNA-seq from 4596 cells isolated from dissected border zone regions of regenerating zebrafish hearts at 7 dpci. The microdissected region of 7 dpci ventricles used for scRNA-seq is depicted in the upper left. Created in BioRender. Beisaw, A. (2025) https://BioRender.com/zek72tx. **B** Gene ontology (GO) analysis of biological processes (BP) in differentially expressed genes enriched in BZ CMs compared to rCMs. log_10_padj was calculated in comparison to all genes in the zebrafish genome using Fisher’s exact test and corrected for multiple comparisons using the Bonferroni method. log_size corresponds to the log_10_(number of annotations for the GO Term ID in zebrafish from the EBI GOA Database). **C** Differentially expressed genes (padj < 0.05) arising from pseudo-bulk comparisons of gene expression in rCM versus BZ CMs (left) and mac2 versus mac3 (ECM, right) clusters. *P*-values were calculated using unpaired two-sided *t*-tests and corrected for multiple comparisons using the Benjamini-Hochberg method. **D** Violin plots of expression of genes involved in actin cytoskeleton and CM regulation in rCMs vs. BZ CMs clusters from scRNA-seq at 7 dpci. **E** Schematic illustrating the experimental set-up in *Tg(myl7:Cre-ERT2); Tg(ubb:loxP-EGFP-loxP-AFos-P2A-tagBFP)* (CM:*AFos*, top). Control CMs (*n* = 3 independent pools of 6 ventricles each) and CM:*Afos* CMs (*n* = 3 independent pools of 6 ventricles each) were isolated at 7 dpci and RT-qPCR analysis of actin cytoskeletal and ECM regulators enriched in BZ CMs was performed. Data are presented as mean ± SD. *P*-values were calculated using unpaired two-sided *t*-tests or a two-sided Mann–Whitney test (*pfn1*). Source data are presented in the Source Data file. Created in BioRender. Beisaw, A. (2025) https://BioRender.com/qqhzxg5. **F** Immunostaining of F-actin and Vinculin in control and CM:*A-Fos* ventricles at 7 dpci. Yellow arrowheads denote protruding CMs with Vinculin enrichment at the leading edge of protrusions. **G** Violin plots of expression of genes involved in ECM composition/regulation in mac3 (ECM) vs. mac2 clusters from scRNA-seq at 7 dpci. Scale bars: 20 μm.

In order to find potential regulators of CM protrusion, we performed dissection of the wound border zone at 7 dpci and subjected these cells to single-cell RNA-sequencing (scRNA-seq, Fig. 6A). After removal of single cell transcriptomes that did not pass our quality control metrics (Supplementary Fig. 5A–C), 4596 cells remained. Dimensionality reduction with UMAP revealed 12 clusters of cells (Fig. 6A). Dot plots of unique markers for each cluster and for known cell type-specific marker genes verified the presence of known cell types at the border zone (Supplementary Fig. 5D, E). 36.3% of cells from our scRNA-seq dataset were CMs, including remote CMs (rCM) and border zone CMs (BZ CM), expressing previously described border zone marker genes, such as *mustn1b*, *tagln*, and *myl6*^40,54^. 32.6% of cells from our scRNA-seq were fibroblasts, expressing known fibroblast genes, such as *col1a2, col1a1a, postnb*, and *fn1b*. We also observed specific fibroblast clusters, including Fb1 (*acta2*+), Fb2 (*c4* and *cxcl12a*+), a recently described regenerative Fb3 cluster (*col12a1a*+)^23^, and a small Fb4 (*fosab*+) cluster. 14.9% of cells from our scRNA-seq were endocardial/endothelial cells, including cluster EC1 (fibroblast-like EC (fEC) expressing endocardial markers, such as *fli1a* and *sele*, and fibroblast markers, such as *nppc* and *spock3*)^23^ and cluster EC2, expressing more traditional endothelial markers (eEC), including *kdrl* and *cldn5b*. The remaining 15.6% of cells from our scRNA-seq dataset were comprised of 4 immune/macrophage clusters, including mac1 (expressing *apoeb* and *wt1b*)^34^, mac2 (expressing canonical macrophage markers such as *mpeg1.1*, *mfap4*, *csfr1a, c1qa* and *grap2b*), mac3 (ECM, expressing a number of macrophage markers, including *mpeg1.1* and *mfap4*, in addition to fibroblast-like genes, such as *postnb, col1a2, fn1b*, and *col12a1a*), and a small cluster of myeloid-derived cells (MDC, expressing beta-like 1 defensin (*defbl1*) and small integral membrane protein 29 (*smim29*)) (Supplementary Fig. 5D, E).

In order to investigate molecular mechanisms underlying CM protrusion, we performed differential gene expression and gene ontology analyses between the BZ CM and remote CM clusters. As expected, differentially expressed genes (DEGs) that were higher in the remote CMs were enriched for biological processes such as ‘regulation of heart contraction’ and ‘generation of precursor metabolites and energy’, and included genes such as *myl7, pln*, and *ttn.2*, and genes encoding proteins involved in fatty acid oxidation (*rbp2a, hadh*, and *acadm*), cellular respiration and mitochondrial electron transport (*coq10b* and *sdha*), and the tricarboxylic acid (TCA) cycle (*mdh1ab*) (Supplementary Fig. 6A, B). DEGs that were higher in border zone CMs included known border zone markers (*mustn1b*, *tagln, myh7, myl6*, and *nppb*), along with genes that were enriched for biological processes such as ‘regeneration’ (*mdka, ccn1*, and *ccn2a*) and ‘actin cytoskeleton organization’ (*sdc4, pfn1, cfl1, rhoab*, and *arpc3*) (Fig. 6B–D and Supplementary Fig. 6C, D). Furthermore, we observed an enrichment of AP-1 transcription factor (TF) family members *jun, junba, junbb, fosab*, and *fosl1a* in border zone CMs, in line with our previously published data that AP-1 TFs are enriched in BZ CMs in response to cryoinjury^43^. Notably, Reactome pathway analysis of DEGs enriched in BZ CMs revealed pathways such as ‘cell:ECM interactions’, ‘collagen biosynthesis and modifying enzymes’, and ‘collagen degradation’ (Supplementary Fig. 6E). The expression of genes, such as *col1a1a, col1a2, col6a2, pcolce2b, fn1a*, *itga5, tln1*, and *timp2a* were enriched in a subcluster of BZ CMs compared to remote CMs (Fig. 6D and Supplementary Fig. 6F). In order to determine whether these gene expression programs were disrupted in models that display defects in CM protrusion, we utilized a genetic model to block AP-1 transcription factor function in CMs, where we previously observed a significant decrease in CM protrusion number and length into the injured tissue^43^. Isolation of CMs from *Tg(myl7:Cre-ERT2); Tg(ubb:loxP-EGFP-loxP-AFos-P2A-tagBFP)* (hereafter referred to as CM:*A-Fos*) ventricles at 7 dpci and RT-qPCR of a panel of genes whose protein products regulate the actin cytoskeleton and ECM revealed a decrease in expression of *pfn1* and *pfn2* (regulators of actin polymerization) and *pcolce2b* (a collagen binding protein that has recently been shown to inhibit Bmp1/tolloid-like protease-mediated collagen maturation^55^) (Fig. 6E). Furthermore, we investigated the localization of the focal adhesion protein Vcl, based on enrichment of *tln1* in BZ CMs and our previously published data showing BZ CM localization of the focal adhesion-enriched protein integrin-linked kinase (Ilk)^43^. We found that Vcl localizes to the leading edge of CM protrusions at the BZ in control hearts at 7 dpci and blocking AP-1 function in CMs leads to a decrease in Vcl levels in CM protrusions (Fig. 6F). Altogether, these data suggest that CMs upregulate gene expression programs that regulate actin cytoskeleton organization, cell:ECM interaction, and the ECM/collagen environment at the border zone to promote the migratory behavior we observe in our live-imaging data. Furthermore, our analyses of CM:*A-Fos* hearts places AP-1 upstream of several regulators of actin cytoskeleton, cell:ECM interaction, and the ECM/collagen environment in regenerating zebrafish CMs.

We next focused on the 4 immune cell clusters that arose in our scRNA-seq analysis. mac2 (*grap2b*+) and mac3 (ECM) expressed several canonical macrophages markers, including *mpeg1.1, mfap4, spi1b, c1qa, marco, ccl34a.4*, and *coro1a* (Supplementary Fig. 7A). Furthermore, mac2 and mac3 clusters expressed a number of alternatively-activated macrophage markers, including *mrc1b* and *tgm2b*, and *tcima*, expressed in a recently described population of macrophages that stimulate phagocytosis during zebrafish spinal cord regeneration^56^ (Supplementary Fig. 7A). Cells within cluster mac1(*apoeb*+) exhibited high expression of *apoeb*, encoding for Apolipoprotein E, whose expression in hepatocytes and macrophages is known for its key protective role in atherosclerosis^57^. In zebrafish, *apoeb* has been described as a marker of microglia^58^, and has been described in macrophage subpopulations in the heart^30^ and in a sensory hair cell regeneration model in larvae^59^. Lastly, a small cluster of myeloid-derived cells (MDC) was observed that highly expressed beta-like defensin 1 (*defbl1*), a molecule involved in the innate immune response to bacteria, myeloid associated differentiation marker (*MYADM/myadma*), monocyte-enriched *gpnmb*, and genes expressed in granulocytes, including *cd9b* and *smim29*. Notably, there was no detection of neutrophil-enriched genes, including *mpx* or *lyz*, in any cells from our scRNA-seq dataset.

Based on our observations that macrophages are essential for CM protrusion length and for collagen degradation/remodeling at the border zone (Figs. 4C and 5A, B), we focused our attention on the mac3 (ECM) cluster in our scRNA-seq dataset. Differential gene expression and gene ontology analyses between mac3 (ECM) and mac2 (expressing canonical macrophage markers) revealed an enrichment in biological processes such as ‘extracellular matrix organization’ (*col1a2, mmp14b, loxl2b, fn1a, col4a1*, and *col5a1*), ‘cell migration’ (*cxcl12a, rab13, cxcl18b, rnd3b*, and *fn1a*), and ‘regeneration’ (*yap1, aldh1a2, mycb, ccn1*, and *anxa1a*), and genes were enriched in the mac3 (ECM) cluster that were involved in ‘collagen degradation’ (*mmp2, mmp14a*, and *mmp14b*), and ‘collagen biosynthesis and modifying enzymes’ (*col12a1a, pcolce2b, col4a2*, and *col6a3*) (Figs. 6C, G and Supplementary Fig. 7B, C). Taken together, our scRNA-seq analyses reveal regulation of the ECM and ECM remodeling by border zone CMs and macrophages, which contributes to CM protrusion into the injured cardiac tissue during regeneration.

### *mmp14b* expression is restricted to the border zone

**Fig. 7:**
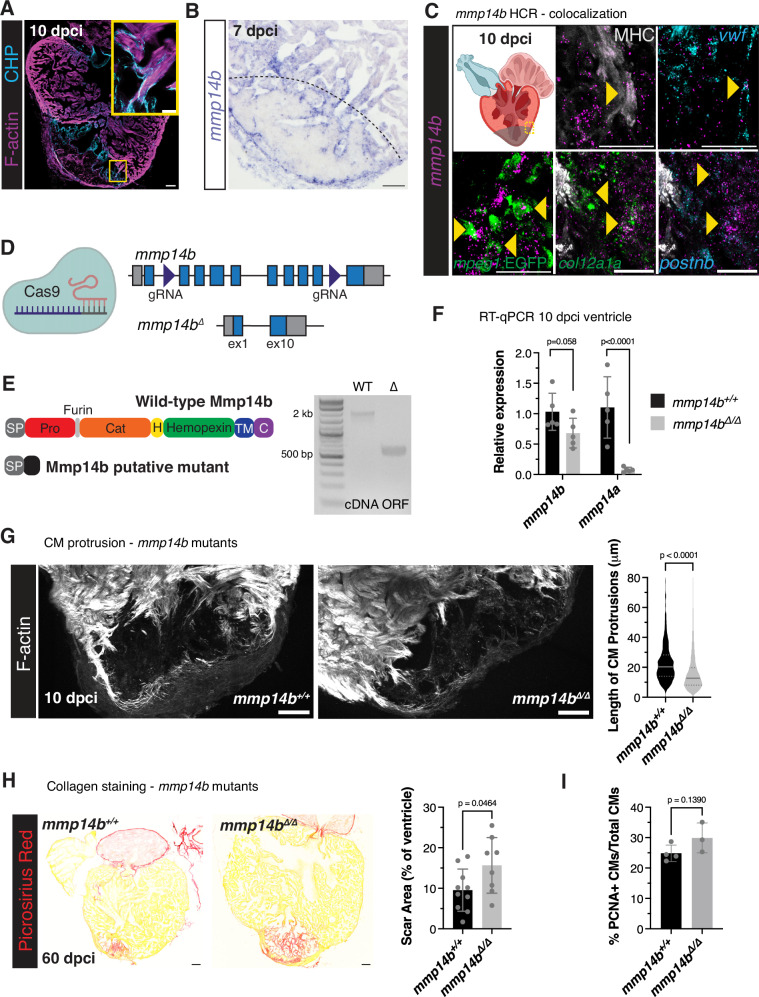
Mmp14b is a regulator of CM protrusion. **A** Collagen hybridizing peptide (CHP) and phalloidin staining of a wild-type ventricle at 10 dpci. Yellow box denotes the area in the zoomed image. **B** In situ hybridization of *mmp14b* expression in a wild-type ventricle at 7 dpci. Black dashed line denotes the approximate injury border. **C** Colocalization of *mmp14b* (HCR, magenta) with CMs (MHC immunostaining), endocardial cells *(vwf* HCR), macrophages (GFP immunostaining in *Tg(mpeg1:EGFP)* ventricles, and fibroblasts (*col12a1a* (HCR, green) and *postnb* (HCR, cyan) in the cortical BZ region at 10 dpci. Yellow box in the schematic of the heart marks the cortical CM region depicted in the zoomed images. Created in BioRender. Beisaw, A. (2025) https://BioRender.com/zek72tx. **D** Schematic depicting the exon structure of the *mmp14b* locus and CRISPR/Cas9-induced full-length deletion between exons 2 and 9 of *mmp14b*. Created in BioRender. Beisaw, A. (2025) https://BioRender.com/pepiu5b. **E** Mmp14b wild-type and putative mutant protein domain structure (left). RT-PCR of the *mmp14b* open reading frame from wild-type and *mmp14b*^*Δ/Δ*^ mutant embryos (right). SP signal peptide, Pro propeptide, Cat catalytic domain, H hinge region, TM transmembrane domain, C C-terminal tail. **F** RT-qPCR of *mmp14b* and *mmp14a* expression in single ventricles from *mmp14b*^*Δ/Δ*^ (*n* = 5 ventricles) and wild-type siblings (*n* = 5 ventricles) at 10 dpci. Data are presented as mean ± SD. *P*-values were calculated using an unpaired two-sided *t*-test. Source data are presented in the Source Data file. **G** Phalloidin staining of thick cryosections from *mmp14b*^*Δ/Δ*^ and wild-type sibling ventricles at 10 dpci (left). Quantification of CM protrusion length (right, *mmp14b*^*Δ/Δ*^ *n* = 1124 CM protrusions from 8 ventricles, wild-type sibling *n* = 1480 CM protrusions from 9 ventricles). Data are presented as violin plots of all points with solid gray lines indicating the median and dotted gray lines indicating 25th and 75th percentile. *P*-values were calculated using a two-sided Mann–Whitney test. Source data are presented in the Source Data file. **H** Picrosirius red staining of collagen in *mmp14b*^*Δ/Δ*^ (*n* = 8 ventricles) and wild-type sibling (*n* = 10 ventricles) at 60 dpci (left). Quantification of scar area (% of ventricle area) on the right. Data are presented as mean ± SD. *P*-value was calculated using an unpaired two-sided *t*-test. Source data are presented in the Source Data file. **I** Quantification of CM proliferation within 100 μm of the wound border from PCNA/Mef2 immunostaining in *mmp14b*^*Δ/Δ*^ (*n* = 3 ventricles) and wild-type sibling (*n* = 4 ventricles) at 7 dpci. Data are presented as mean ± SD. *P*-value was calculated using an unpaired two-sided *t*-test. Source data are presented in the Source Data file. Scale bars: 100 μm in (**A**, **B**, **G**, and **H**), 20 μm in zoomed image in (**A** and **C**).

We were intrigued by the presence of subclusters of CMs and macrophages at the border zone expressing several collagen genes and genes that are involved in collagen modification and degradation. Collagen hybridizing peptide (CHP) staining, marking remodeling/degrading collagen, and incubation of fresh sections with caged DQ-Collagen, fluorescently marking collagenase activity, revealed association of collagen degradation within the injured area, in line with previously published data^60^, and with border zone CMs at 10 dpci (Fig. 7A and Supplementary Fig. 8A). Mining of previously published tomo-seq data from regenerating zebrafish hearts^40^ revealed an enrichment in *mmp14b* expression at the border zone, which we confirmed by in situ hybridization (Fig. 7B). Mmp14b is a membrane-tethered matrix metalloproteinase with multiple known ECM substrates^61^, including type I, II, and III collagens^62^, gelatin, fibronectin, and fibrin^63^, as well as non-ECM substrates, including pro-MMP2^64^, pro-MMP13^65^, and ADAM9^66^. Furthermore, MMP14 has been associated with cell migration in multiple contexts and has been observed in invadopodia, protrusive cellular structures found in invasive cells that promote cell attachment to and degradation of ECM^67^. *mmp14b* expression was upregulated in regenerating zebrafish hearts at 7 and 10 dpci (Supplementary Fig. 8B) and enriched in multiple cell types from our scRNA-seq data, including BZ CMs, macrophages (mac1 and mac3(ECM)), endocardial cells (EC2), and fibroblasts (Fb2, Fb3, and Fb4) (Supplementary Fig. 8C). Accordingly, sorting of CMs and macrophages by FACS revealed expression of *mmp14b* at 7 dpci and hybridization chain reaction (HCR) RNA-FISH revealed colocalization of *mmp14b* and CM and macrophage markers at the border zone at 10 dpci (Fig. 7C and Supplementary Fig. 8D, E). Furthermore, we observed CM-specific and injury-responsive regions of accessible chromatin in intron 1 of the *mmp14b* locus from our previously published ATAC-seq data^43^, which differed from a recently described endothelial-specific *mmp14b* enhancer region from regenerating zebrafish hearts (Supplementary Fig. 8F)^68^.

We hypothesized that Mmp14b plays a role in remodeling collagen/the ECM environment at the border zone to regulate CM invasion. To test this hypothesis, we generated full locus deletion *mmp14b* mutants using CRISPR/Cas9 technology and 2 gRNAs flanking exon 2 and exon 9 of the *mmp14b* gene (Fig. 7D). Genotyping of larvae from a heterozygous *mmp14b*^*Δ/+*^ incross led to Mendelian ratios of wild-type, heterozygous, and mutant larvae at 24 hpf (Supplementary Fig. 9A). Deletion of *mmp14b* between exons 2–9 likely leads to an intact 5’ end of the Mmp14b protein with 29 amino acids (aa) containing the signal peptide and a frameshift consisting of 18 aa before a premature stop codon from exon 10 and presence of a shortened mutant *mmp14b* transcript in *mmp14b*^*Δ/Δ*^ deletion mutant larvae was detected (Fig. 7E). We observed no gross morphological phenotypes during embryonic and larval development of *mmp14b*^*Δ/Δ*^ mutants compared to wild-type siblings and *mmp14b*^*Δ/Δ*^ mutants survived to adulthood. Based on previously published observations that single CRISPR/Cas9-induced mutations in *mmp14b* leads to nonsense mediated decay (NMD) and upregulation of the paralogous *mmp14a*^69^, we used RT-qPCR to assess levels of *mmp14a* and *mmp14b* in *mmp14b*^*Δ/Δ*^ deletion mutants. In *mmp14b*^*Δ/Δ*^ mutant hearts at 10 dpci, we observed a slight decrease in *mmp14b* mutant transcript levels, and *mmp14a* expression was significantly downregulated (Fig. 7F). This dramatic decrease in *mmp14a* levels in the *mmp14b*^*Δ/Δ*^ mutants at 10 dpci was specific to the regenerating heart, as we only observed a slight decrease in *mmp14a* in *mmp14b*^*Δ/Δ*^ mutants during larval stages (Supplementary Fig. 9B). These results suggest that there is no genetic compensation from *mmp14a* in the *mmp14b*^*Δ/Δ*^ full length deletion mutants.

### Mmp14b regulates ECM remodeling and CM protrusion

In order to determine the importance of Mmp14b in CM protrusion during cardiac regeneration, we used F-actin staining to compare CM protrusion number and length in *mmp14b*^*Δ/Δ*^ full length deletion mutants at 10 dpci. We found that while there was no difference in the number of CM protrusions (Supplementary Fig. 9C), we observed a significant decrease in the length of CM protrusions at the border zone in *mmp14b*^*Δ/Δ*^ mutants when compared to wild-type siblings (Fig. 7G), similar to our observations in *irf8* mutant ventricles lacking macrophages. Using Picrosirius red staining, we found that *mmp14b*^*Δ/Δ*^ mutants exhibited defects in cardiac regeneration, with the significant presence of collagen-containing scar tissue at 60 dpci when compared to wild-type siblings (Fig. 7H). These defects in cardiac regeneration were not due to differences in CM proliferation, as CM proliferation was not affected in the *mmp14b*^*Δ/Δ*^ mutants generated here when compared to wild-type siblings at 7 dpci (Fig. 7I).

**Fig. 8:**
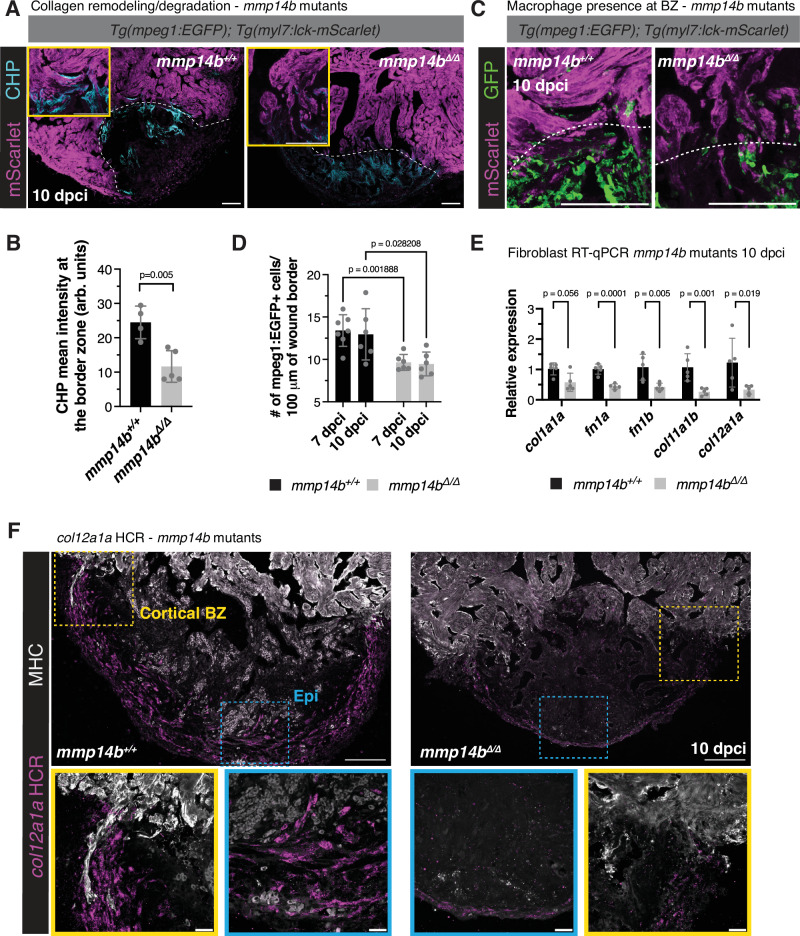
Mmp14b is essential for macrophage presence and ECM remodeling at the border zone. **A** Collagen hybridizing peptide (CHP) and mScarlet immunostaining in *Tg(mpeg1:EGFP); Tg(myl7:lck-mScarlet)* ventricles from *mmp14b*^*Δ/Δ*^ and wild-type siblings at 10 dpci. White dashed lines indicate the wound border and yellow boxes contain zoomed images from the wound border zone. **B** Quantification of CHP intensity at the border zone (arb. units, arbitrary units) in *Tg(mpeg1:EGFP); Tg(myl7:lck-mScarlet)* ventricles from *mmp14b*^*Δ/Δ*^ (*n* = 5 ventricles) and wild-type siblings (*n* = 4 ventricles) at 10 dpci. Data are presented as mean ± SD. *P*-value was calculated using an unpaired two-sided *t*-test. Source data are presented in the Source Data file. **C** GFP and mScarlet immunostaining in *Tg(mpeg1:EGFP); Tg(myl7:lck-mScarlet)* ventricles from *mmp14b*^*Δ/Δ*^ and wild-type siblings at 10 dpci. White dashed lines indicate the approximate wound border. **D** Quantification of *mpeg1*:EGFP+ cells 50 μm proximal and distal to the wound border in ventricles from *mmp14b*^*Δ/Δ*^ (*n* = 6 ventricles) and wild-type siblings (7 dpci *n* = 7 ventricles, 10 dpci *n* = 6 ventricles) at 7 and 10 dpci. Data are presented as mean ± SD. *P*-values were calculated using unpaired two-sided *t*-tests and corrected for multiple comparisons using the Holm-Sidak method. Source data are presented in the Source Data file. **E** RT-qPCR analysis of fibroblast marker genes in *mmp14b*^*Δ/Δ*^ mutant (*n* = 5 ventricles) and wild-type sibling (*n* = 5 ventricles) at 10 dpci. Data are presented as mean ± SD. *P*-values were calculated using an unpaired two-sided *t*-test or a two-sided Mann–Whitney test (*col1a1a*). Source data are presented in the Source Data file. **F** *col12a1a* in situ hybridization chain reaction (HCR) in *mmp14b*^*Δ/Δ*^ mutant and wild-type sibling ventricles at 10 dpci. Yellow boxes denote zoomed images at the cortical BZ, blue boxes denote zoomed images at the apex of the wound. Epi, epicardium. Scale bars: 100 μm, 20 μm in the zoomed images in (**F**).

Due to the similarity in CM protrusion phenotypes between *mmp14b*^*Δ/Δ*^ and *irf8*^*st96/st96*^ mutants, we used collagen hybridizing peptide (CHP) staining to assess ECM degradation/remodeling at the border zone. Unlike *irf8*^*st96/st96*^ mutants, *mmp14b*^*Δ/Δ*^ mutants exhibited CHP staining within the wound at 10 dpci (Fig. 8A). However, quantification of CHP staining directly at the border zone revealed a significant decrease in CHP intensity in *mmp14b*^*Δ/Δ*^ mutants compared to wild-type siblings at 10 dpci (Fig. 8B). Notably, this decrease in CHP intensity was accompanied by a corresponding decrease in *mpeg1:*EGFP+ cells at the border zone in *mmp14b*^*Δ/Δ*^ mutants compared to wild-type siblings (Fig. 8C, D). These observations are in line with those from *irf8*^*st96/st96*^ mutants, which lack macrophages, and with published data reporting a decrease in macrophage number proximal to the wound border at 3 days post apical resection in zebrafish hearts treated with a small molecule MMP14 inhibitor^70^. Taken together, these results indicate that Mmp14b is important for macrophage presence and ECM degradation/remodeling at the wound border to promote CM protrusion.

As our scRNA-seq data revealed the expression of *mmp14b* in multiple cell types, including endothelial/endocardial cells and fibroblasts (Supplementary Fig. 8C), we addressed the potential contribution of these cell types to the observed CM protrusion defects in *mmp14b*^*Δ/Δ*^ mutants. First, we performed immunostaining with an antibody for Aldh1a2, which has previously been shown to mark activated endocardium and epicardium^19^, in *mmp14b*^*Δ/Δ*^ mutants and wild-type siblings at 7 dpci. We observed no significant difference in Aldh1a2 levels or expression pattern in both *mmp14b*^*Δ/Δ*^ mutants, and *irf8*^*st96/st96*^ mutants that largely lack macrophages, when compared to wild-type (Supplementary Fig. 10A). We confirmed these results with an additional transgenic endocardial marker, *sqET33-1Aet* (hereafter referred to as *ET33-1a*), and similarly found no significant difference in endocardial presence in the wound in *mmp14b*^*Δ/Δ*^ mutants and *irf8*^*st96/st96*^ mutants at 7 dpci (Supplementary Fig. 10B).

We used RT-qPCR of a panel of fibroblast markers to determine whether *mmp14b* deletion affects fibroblast gene expression in response to cryoinjury. We found no significant differences in expression of canonical fibroblast markers, including *postnb, acta2*, and *vim*, or endocardial-derived fibroblast markers, *nppc* and *spock3*, in *mmp14b*^*Δ/Δ*^ mutant compared to wild-type sibling ventricles at 10 dpci (Supplementary Fig. 10C). However, we observed a significant decrease in expression of regenerative fibroblast markers, *col12a1a* and *col11a1b*, and ECM components, including *col1a1a, fn1a*, and *fn1b*, in *mmp14b*^*Δ/Δ*^ mutant compared to wild-type sibling ventricles at 10 dpci (Fig. 8E). In situ hybridization chain reaction confirmed *col12a1a* expression in epicardial-derived fibroblasts surrounding the injured area and high expression surrounding cortical CM protrusion in wild-type ventricles at 10 dpci (Fig. 8F). *Col12a1a*-expressing cells at the cortical border zone were in close proximity to macrophages (Supplementary Fig. 10D), and a significant decrease in *col12a1a* expression was observed in *mmp14b*^*Δ/Δ*^ mutant compared to wild-type sibling ventricles (Fig. 8F). These data suggest that Mmp14b is important for the presence of a regenerative population of fibroblasts following cryoinjury. While these Col12a1a+ regenerative fibroblasts localize on the apical surface of the cryoinjured zebrafish heart at 7 dpci^23,71^ and 10 dpci (Fig. 8F and Supplementary Fig. 10E), and are therefore not in direct contact with trabecular protruding cardiomyocytes at the border zone, we cannot exclude fibroblast contribution to the *mmp14b* mutant phenotype that we observe.

### CM-specific *mmp14b* overexpression can promote CM protrusion

**Fig. 9:**
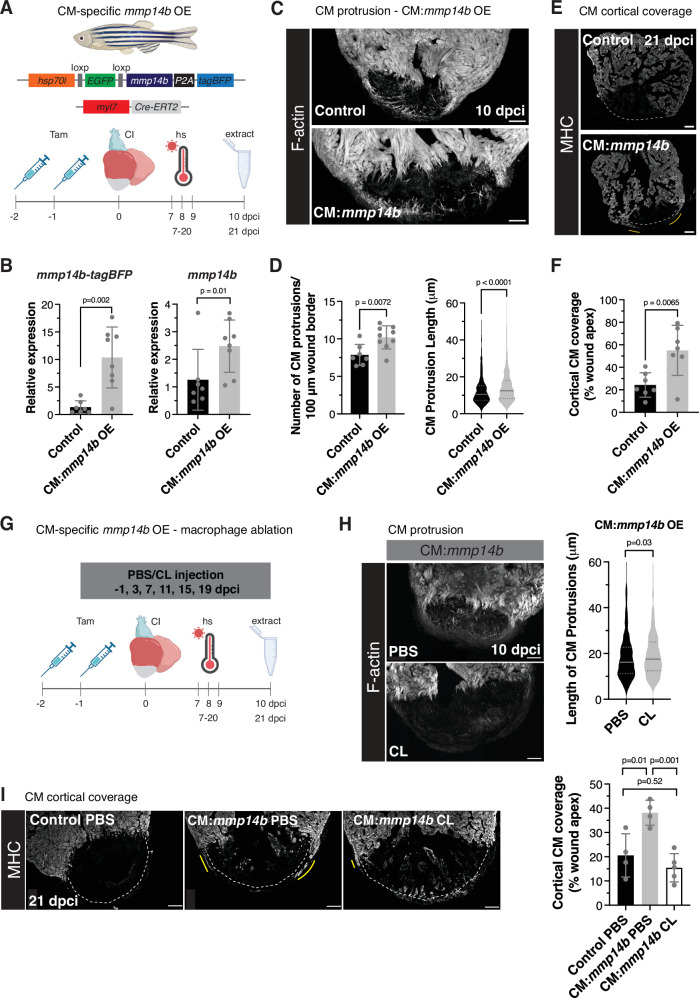
CM-specific overexpression of *mmp14b* promotes CM protrusion. **A** Schematic illustrating the *Tg(hsp70l:loxP-EGFP-loxP-mmp14b-P2A-tagBFPHA); Tg(myl7:Cre-ERT2)* line and experimental scheme to induce CM-specific overexpression of *mmp14b*. Created in BioRender. Beisaw, A. (2025) https://BioRender.com/ifqm2g8. **B** RT-qPCR of *mmp14b-P2A-tagBFP* (left) and *mmp14b* (right) in control (*n* = 7 ventricles) and CM:*mmp14b* OE (*n* = 8 ventricles) zebrafish at 10 dpci. Data are presented as mean ± SD. *P*-values were calculated using unpaired two-sided *t*-test (*mmp14b*) or a two-sided Mann–Whitney test (*mmp14b-tagBFP*). Source data are presented in the Source Data file. **C** Phalloidin staining of F-actin in thick cryosections of control and CM:*mmp14b* OE zebrafish ventricles at 10 dpci. **D** Quantification of the number of CM protrusions per 100 microns of wound border and length of CM protrusions from thick cryosections of control (*n* = 814 CM protrusions from 7 ventricles) and CM:*mmp14b* OE (*n* = 1431 CM protrusions from 9 ventricles) zebrafish at 10 dpci. Data are presented as mean ± SD (number of CM protrusions) and violin plots of all points with solid gray lines indicating the median and dotted gray lines indicating 25th and 75th percentile (CM protrusion length). *P*-values were calculated using an unpaired two-sided *t*-test (number of CM protrusions) and a two-sided Mann–Whitney test (CM protrusion length). Source data are presented in the Source Data file. **E** Myosin heavy chain immunostaining of control and CM:*mmp14b* OE ventricles at 21 dpci. White dashed lines denote the wound apex and yellow lines denote cortical CMs that have migrated over the wound apex. **F** Quantification of cortical CM coverage (% wound apex) in control (*n* = 7 ventricles) and CM:*mmp14b* OE (*n* = 7 ventricles) at 21 dpci. Data are presented as mean ± SD. *P*-value was calculated using an unpaired two-sided *t*-test. Source data are presented in the Source Data file. **G** Schematic illustrating the experimental set-up to overexpress *mmp14b* in CMs and ablate macrophages with clodronate liposomes. Created in BioRender. Beisaw, A. (2025) https://BioRender.com/sdatdc8. **H** Phalloidin staining of F-actin in thick cryosections of ventricles from CM:*mmp14b* OE zebrafish injected with PBS control liposomes or clodronate liposomes (CL) at 10 dpci (left). Quantification of the length of CM protrusions from thick cryosections of CM:*mmp14b* OE zebrafish injected with PBS control liposomes (*n* = 598 CM protrusions from 5 ventricles) or clodronate liposomes (CL, *n* = 458 CM protrusions from 3 ventricles)) at 10 dpci (right). Data are presented as violin plots of all points with solid gray lines indicating the median and dotted gray lines indicating 25th and 75th percentile. *P*-values were calculated using a two-sided Mann–Whitney test. Source data are presented in the Source Data file. **I** Myosin heavy chain (MHC) immunostaining of ventricles from control zebrafish injected with PBS liposomes and CM:*mmp14b* OE zebrafish injected with PBS liposomes and clodronate liposomes (CL) at 21 dpci (left). White dashed lines denote the wound apex and yellow lines denote cortical CMs that have migrated over the wound apex. Quantification of cortical CM coverage (% wound apex) in ventricles from control zebrafish injected with PBS liposomes (*n* = 4 ventricles) and CM:*mmp14b* OE zebrafish injected with PBS liposomes (*n* = 4 ventricles) and clodronate liposomes (CL, *n* = 5 ventricles) at 21 dpci (right). Data are presented as mean ± SD. *P*-values were calculated using ordinary one-way ANOVA and Tukey’s multiple comparisons test. Source data are presented in the Source Data file. Scale bars: 100 μm.

In order to determine whether CM-specific *mmp14b* overexpression is sufficient to promote CM invasion, we generated a transgenic line with spatial and temporal control of *mmp14b* overexpression (Fig. 9A). *Tg(hsp70l:loxP-EGFP-loxP-mmp14b-P2A-tagBFP); Tg(myl7:Cre-ERT2)* fish were treated with tamoxifen 2 days prior to cryoinjury (ethanol vehicle was used as a control) to allow for cardiomyocyte-specific *mmp14b* overexpression upon heat shock (Fig. 9A, B). Cardiomyocyte-specific *mmp14b* overexpression (hereafter referred to as CM:*mmp14b* OE) between 7 and 10 dpci led to an increase in the number of CM protrusions and length of those CM protrusions observed at the wound border zone when compared to control ventricles (Fig. 9C, D). Furthermore, Picrosirius red staining to visualize collagen and the wound area at 21 dpci revealed a trend towards decreased wound area in CM:*mmp14b* OE ventricles when *mmp14b* OE was induced from 7–20 dpci as compared to control (Supplementary Fig. 11A). Notably, we observed that CM presence was increased on the apical surface of CM:*mmp14b* OE ventricles compared to control (Fig. 9E, F) at 21 dpci, in a similar location to where we observed highly protrusive cortically-located CMs in our live-imaging analysis (Fig. 2). Altogether, these observations indicate that CM-specific *mmp14b* overexpression is sufficient to induce CM protrusion and invasion of collagen-rich injured tissue after cardiac cryoinjury.

In order to determine whether CM:*mmp14b* OE was sufficient to promote CM invasion in the absence of ECM remodeling at the border zone when macrophages were absent, we injected CM:*mmp14b* OE fish with clodronate liposomes 1 day prior to cryoinjury and every 4 days afterwards. We then induced CM:*mmp14b* OE using heat shock at 7–9 dpci and measured CM protrusion at 10 dpci, or 7–20 dpci and measured cortical CM coverage at 21 dpci (Fig. 9G). At 10 dpci, we found that macrophage depletion with CL did not affect the ability of CM:*mmp14b* OE to increase CM protrusion number and length (Fig. 9H and Supplementary Fig. 11B). However, depletion of macrophages with CL significantly reduced cortical CM coverage of the wound in CM:*mmp14b* OE ventricles at 21 dpci to levels of control ventricles (Fig. 9I). These results suggest that there may be differences in the function or requirement for macrophages in CM invasion in the trabecular versus cortical border zone of the regenerating zebrafish heart.

## Discussion

Our data provide the first comprehensive analysis of CM protrusion into the injured collagenous tissue during the process of zebrafish heart regeneration. We found a peak in CM protrusion number and length between 7 and 10 dpci. We found that nearly all border zone CMs that extend protrusions into the injured tissue have undergone dedifferentiation based on the expression of several published dedifferentiation markers and the disassembly of their sarcomeres. Furthermore, we observed that the majority of CMs that extend protrusions at the border zone are not actively proliferating based on a CM-specific FUCCI reporter line. While we hypothesize that CM protrusion and invasion occur downstream of dedifferentiation and proliferation, we do not yet have the tools to understand whether all protruding CMs at the border zone have proliferated or whether remaining/spared CMs after injury can invade without dedifferentiating and proliferating. Such investigation requires a lineage tracing approach of cells that have undergone cell cycle/division, which, to the best of our knowledge, has not yet been reported in zebrafish.

**Fig. 10:**
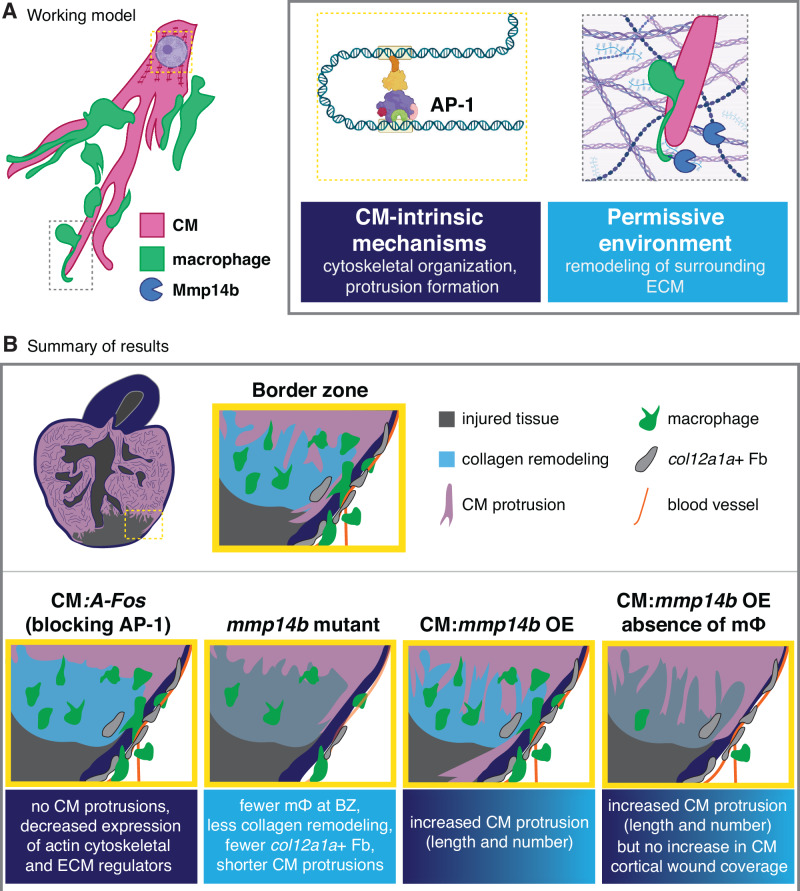
Working model and summary of our study. **A** Working model of CM invasion to replace injured tissue during zebrafish heart regeneration. CM-intrinsic mechanisms, partly regulated by AP-1 transcription factors, promote actin cytoskeletal remodeling/organization and protrusion formation, while Mmp14b and macrophages contribute to remodeling the ECM at the border zone to create a permissive environment for CM invasion. Created in BioRender. Beisaw, A. (2025) https://BioRender.com/19dzxy0. **B** Summary of the genetic models presented in our study and their effects on CM protrusion and invasion, including CM:*A-Fos* to block AP-1, *mmp14b* deletion, CM:*mmp14b* OE, and CM:*mmp14b* OE in the absence of macrophages. The effects of *mmp14b* deletion on endothelial cells (blood vessels) in the model is from previously published data^68^. BZ border zone, CM cardiomyocyte, ECM extracellular matrix, Fb fibroblast, mϕ macrophage, OE overexpression.

Through live-imaging of regenerating ventricular slices and scRNA-seq analyses, our results suggest that border zone CMs exhibit characteristics of actively migrating cells, displaying extension of motile filopodia directed towards the injured tissue and upregulation of gene expression programs that promote cell migration and remodel the ECM. We cannot yet conclude with certainty that these gene expression programs and motile filopodia extension result in migration of these cells to replace the injured tissue; as the process of CM invasion is relatively slow, development of ex vivo methods to track CM protrusions over a period of days is required to definitively track movement of cardiomyocytes and correlate CM protrusion dynamics with CM invasion. We have previously published that AP-1 transcription factors are cell-autonomous regulators of CM protrusion and that blocking AP-1 activity results in a dramatic reduction in CM protrusion at the wound border zone^43^. Here, we further show that blocking AP-1 activity in CMs leads to a decrease in the expression of a subset of genes whose protein products regulate actin cytoskeletal organization and the ECM (Fig. 10A, B). This cell-autonomous regulation is in line with studies from adult mouse models of cardiomyocyte regeneration that exhibit protrusion of CMs: in Hippo signaling-deficient mouse CMs, YAP binds to a number of genomic targets near genes that regulate actin cytoskeletal dynamics and cell:ECM interaction^10^, and CM-specific constitutively active *Erbb2* overexpression leads to the upregulation of both epithelial-to-mesenchymal transition (EMT) and ECM remodeling genes^15^. These data suggest that CM-intrinsic genetic programs that promote cell migration and invasion are upregulated in regenerating CMs and are important to replenish injured collagen-containing tissue with newly formed CMs (Fig. 10A).

We provide evidence that macrophages are closely associated with protruding/invading CMs and regulate the CM migration process through remodeling of ECM at the border zone. Furthermore, through resident macrophage predepletion experiments, our data indicate that the resident macrophage population is at least partly responsible for this ECM remodeling process. Through scRNA-seq analysis of border zone cells, we find a population of macrophages that express genes important for the remodeling and degradation of ECM, in line with recently published studies that independently corroborate the presence of this ECM macrophage cluster in the regenerating zebrafish heart^31,72^. Notably, it has been recently shown that the resident macrophage population is essential for cardiac regeneration in zebrafish^30^, and that resident macrophage populations in the neonatal and adult mouse are essential for improving functional output following multiple types of cardiac injury and disease^73–75^. We suggest that one function of the resident macrophage population after cardiac injury is to promote CM protrusion and replenishment of collagen-containing injured tissue with CMs. Likely, both macrophage-mediated remodeling of the ECM and CM-specific gene expression programs are necessary for efficient replenishment of the injured tissue with functional cardiomyocytes (Fig. 10A). Whether macrophages play instructive roles for CM invasion in addition to their regulation of ECM remodeling remains to be determined.

We show that Mmp14b is essential for macrophage presence and ECM remodeling at the wound border zone and that CM-specific *mmp14b* overexpression is sufficient to further promote CM invasion in regenerating zebrafish hearts (Fig. 10B). These observations are in line with a previously published study showing that MMP14 inhibition with a small molecule inhibitor leads to decreased macrophage presence near the wound border zone in an apical resection model of cardiac injury in zebrafish^70^. A recent study has also shown that Mmp14b is an important regulator of scar resolution during zebrafish cardiac regeneration, in line with our data here^68^. However, both of these previous studies observed defects in CM proliferation that likely contribute to the defect in cardiac regeneration in response to pharmacological MMP14 inhibition (both Mmp14a and Mmp14b) and *mmp14b* mutation, which we did not observe with the *mmp14b* mutants generated here. We hypothesize that the discrepancy in CM proliferation phenotypes may arise due to: (i) use of a pharmacological inhibitor that targets both Mmp14a and Mmp14b and potential off-target effects of drug treatment, or (ii) differences in cell composition, or in the regeneration process, in apical resection versus cryoinjury models, as both previous studies were performed using apical resection in zebrafish. Previously published meta-analysis comparing transcriptomic data of whole ventricles from apical resection, genetic ablation of CMs, and cryoinjury revealed a core set of regeneration genes that were shared among different injury models, as well as injury-specific gene expression programs^76^. Furthermore, transcriptomic analysis of isolated macrophages from injured zebrafish hearts revealed differences in the temporal dynamics of gene expression in apical resection versus cryoinjury^31^. While these studies suggest that differences in the regeneration process occur in response to various injury models, there has not yet been a systematic study comparing cell composition and differences in the cardiac regeneration process in resection versus cryoinjury. Analysis of previously published scRNA-seq data in apically resected and cryoinjured hearts at multiple timepoints following injury could be useful to compare the regenerative response in these injury models^23,77^. Our data indicate that there are likely differences in the requirement for Mmp14b in stimulating CM proliferation in response to apical resection and cryoinjury.

As mentioned above, Zlatanova and colleagues recently reported that *mmp14b* is expressed and plays an important role in endothelial cells following apical resection^68^. Deletion of an endothelial-specific enhancer of *mmp14b* leads to decreased *mmp14b* expression following injury and defects in CM proliferation and scar resolution in resected zebrafish ventricles, indicating that Mmp14b function in endothelial cells is essential for cardiac regeneration. In the *mmp14b* full deletion mutants generated here, we find that Aldh1a2 (marker of activated endocardium and epicardium) and endocardial GFP (marked with the *ET33-1a* enhancer trap line) localization and intensity in the wound appear unchanged, suggesting that Mmp14b is not essential for endocardial presence in the injured tissue. However, we cannot rule out that Mmp14b function in endothelial/endocardial cells contributes to the CM protrusion phenotypes observed in the *mmp14b* mutants generated here.

We find that *mmp14b* is expressed in several cell types following cryoinjury, including fibroblasts, endothelial/endocardial cells, macrophages, and cardiomyocytes at the wound border zone, but it remains unclear if there are cell type-specific functions of Mmp14b in regulating CM protrusion or whether Mmp14b localization at the border zone is sufficient for CM invasion, regardless of the cell source. Our finding that CM-specific OE of *mmp14b* is sufficient to promote CM protrusion and invasion in the trabecular border zone in the absence of macrophages, but not to promote cortical CM coverage of the wound indicates that the requirement for Mmp14b is complex and differs between distinct regions of the heart. These differential effects may be due to the increased number of macrophages and *col12a1a*+ fibroblasts along the surface of the wound (as seen in Figs. 3A and 8F), and their potential importance in promoting cortical CM protrusion and invasion. Additional experiments utilizing genetic models to promote *mmp14b* OE in fibroblasts, endocardial cells, or macrophages would be informative to understand both the regional differences in the heart and the function of Mmp14b in promoting CM invasion. Further, conditional knockout approaches, such as a floxed *mmp14b* allele, or additional tissue-specific *mmp14b* enhancer knockout lines would aid in teasing apart the cell type-specific requirements for Mmp14b in CM invasion.

Our data indicate that genetic deletion of *mmp14b* results in a decrease in the regenerative fibroblast markers *col12a1a* and *fn1a/fn1b*. These results raise multiple questions that warrant further study, especially in light of a recently published study describing a pro-regenerative cellular triad, composed of CMs, macrophages, and fibroblasts, that establish a regenerative microenvironment in adult murine CMs that express YAP^78^. Whether *col12a1a*+ fibroblasts interact with CMs at the wound border, and promote macrophage recruitment to the injured heart, as has been described for *ptx3a*+ fibroblasts in apical resection^79^, is not known. Our data reveal that *col12a1a*+ cells are present nearby macrophages and protruding CMs at the cortical border zone and along the apical surface of the wound. While it is not yet known how regenerative fibroblasts contribute to CM protrusion and invasion of the injured tissue, we hypothesize a likely cooperation between *col12a1a*+ fibroblasts and macrophages in creating a permissive ECM environment to promote CM invasion, especially considering these two cell types are abundant in the region of the heart where we observe highly dynamic CM protrusions. Previously published observations from immunostaining for Col12 in cryoinjured zebrafish hearts reveals Col12 localization on the surface of the wound at 7 dpci and additional Col12 signal within the wound and neighboring border zone CMs at 14 dpci^71^. Whether regenerative *col12a1a*+ fibroblasts migrate into the wound between 7 and 14 dpci, how Mmp14b contributes to this migration, and whether regenerative fibroblasts at 14 dpci contribute to CM protrusion at the trabecular border zone all warrant further study.

Ultimately, we envision that our understanding of zebrafish CM invasion will aid the development of cell-based therapeutic strategies to promote engraftment of CMs into scar tissue to combat heart failure following myocardial infarction. Published studies have shown that while cardiomyocytes derived from human induced pluripotent stem cells (hiPSC) and human ventricular progenitor cells can improve cardiac functional output following cardiac injury, the levels of CM engraftment into fibrotic scar tissue remain relatively low^80–82^. This phenomenon is likely due to multiple factors, including the lack of activation of gene expression programs necessary to stimulate CM invasion and differences in scar composition and stiffness between the regenerative zebrafish and non-regenerative mammalian heart. While fibroblasts are activated and deposit type I collagen in the cryoinjured zebrafish heart, this activation is transient and fibroblasts revert back to an inactivated state during the process of cardiac regeneration^24^. In the mammalian heart, while cardiac fibroblasts are essential for the initial wound healing response and for preventing ventricular rupture^83^, activation of fibroblasts, excessive ECM deposition, and the remaining presence of fibroblasts within the scar lead to a mature and permanent scar^84–86^. Published studies have revealed that decreasing the stiffness of the scar using small molecule compounds can extend the regenerative capacity of the neonatal mouse heart^87^, suggesting that the composition and stiffness of the scar limits regeneration or engraftment of cardiomyocytes. Therefore, it will likely be necessary to both promote invasion of exogenous cardiomyocytes and provide a permissive scar microenvironment when designing cell-based therapeutic strategies to repair the mammalian infarcted heart.

## Methods

All research presented here was performed in accordance with relevant ethical regulations (Max Planck Society and Heidelberg University).

### Zebrafish husbandry and lines

All zebrafish husbandry and experimentation were performed under standard conditions in accordance with institutional (Max Planck Society and Heidelberg University) and national ethical and animal welfare guidelines (RP Karlsruhe and Darmstadt). Lines used in this study include: *Tg(myl7:LIFEACT-GFP)s974*^88^*, Tg(myl7:EGFP)twu26*^89^*, Tg(myl7:mVenus-gmnn)ncv43*^90^*, Tg(myl7:mCherry-cdt1)ncv68*^91^, *Tg(myl7:actn3b-EGFP)sd10*^92^*, Tg(gata4:EGFP)ae1*^93^*, Tg(mpeg1:EGFP)gl22*^94^*, Tg(mpeg1.1:NTR-YFP)w202*^95^*, irf8*^*st96*^^47^, *Tg(cryaa:DsRed, −5.1myl7:CreERT2)pd10*^35^*, Tg(myl7:MKATE-CAAX)sd11Tg*^92^*, sqET331AEt*^96^*, Tg(-0.8mustn1b:EGFP)lim01*^97^*, Tg(ubb:loxP-EGFP-loxP-AFos-P2A-tagBFP)bns335*^43^*, Tg(myl7:lck-mScarlet)bns561* (this study), *mmp14b*^*bns705*^ (this study), and *Tg(hsp70l:loxP-EGFP-loxP-mmp14b-P2A-tagBFP)bns706* (this study), all of which harbored a wild-type AB genetic background. *irf8* mutant fish were genotyped using high-resolution melt analysis (HRMA) with primers described in Supplementary Table 1.

### Generation of the *mmp14b* deletion line using CRISPR/Cas9

CRISPR/Cas9-mediated deletion of the *mmp14b* locus was performed using the Alt-R CRISPR-Cas9 system from Integrated DNA Technologies (IDT). CRISPR RNAs (crRNA) were designed using the Alt-R HDR Design Tool, were annealed to a transactivating crRNA (tracrRNA), and co-injected with Cas9 protein according to the “Zebrafish embryo microinjection” protocol from IDT (contributed by Jeffrey Essner, PhD). 2 gRNAs were co-injected to create a full locus deletion: 5′ gRNA targeting genomic sequence between exon 1 and 2 of *mmp14b* (5′-TACCCCCCAGTTCACCAACTTGG-3′) and exon 9 and 10 of *mmp14b* (5′-TATGACAGGACAATAATGAGAGG-3′). *mmp14b* deletion mutants were genotyped with primers described in Supplementary Table 1.

### Generation of transgenic lines

The 1.5 kb fragment of the *hsp70l* promoter^98^ was PCR amplified and inserted into a pTol2 plasmid backbone. PCR amplification and conventional cloning methods were used to insert a floxed EGFP-stop cassette, the open reading frame of *mmp14b* (amplified from cDNA of 72 hpf zebrafish larvae), a P2A self-cleaving peptide, and HA-tagged tagBFP downstream of the *hsp70l* promoter. 75 pg of *Tol2* mRNA and 30 pg of construct were injected into one-cell stage AB embryos to generate the *Tg(hsp70l:loxP-EGFP-loxP-mmp14b-P2A-tagBFP)* line. For *Tg(myl7:lck-mScarlet)*, *lck* (ATGGGCTGCGTGTGCAGCAGCAACCCCGAG) was inserted upstream of the *mScarlet* ORF via PCR and *lck-mScarlet* was inserted into a pTol2 vector containing the −0.8 kb *myl7* promoter^99^. 75 pg of *Tol2* mRNA and 30 pg of construct were injected into one-cell stage AB embryos to generate the *Tg(myl7:lck-mScarlet)* line.

### Cryoinjury of zebrafish hearts and induction of transgene expression

Cryoinjury was performed according to previously published studies^5–7^. Briefly, adult male and female zebrafish between the ages of 4 and 12 months were anesthetized in 0.025% Tricaine and a small incision was cut in the skin and pericardial sac to expose the heart. The apex of the heart was touched with a liquid nitrogen-cooled metal probe and fish were transferred to a large beaker of system water for recovery.

For the induction of CM-specific *mmp14b* overexpression in *Tg(hsp70l:loxp-EGFP-loxP-mmp14b-P2A-tagBFP); Tg(myl7:CreERT2)* animals, adult zebrafish were anesthetized in 0.025% Tricaine and 0.5 mg/mL of 4-hydroxytamoxifen (4-HT) was injected intraperitoneally for 2 consecutive days. As a negative control, *Tg(hsp70l:loxp-EGFP-loxP-mmp14b-P2A-tagBFP); Tg(myl7:CreERT2)* adult zebrafish were injected with ethanol (EtOH) vehicle for 2 consecutive days. Following IP injection and cryoinjury, transgenic *Tg(hsp70l:loxp-EGFP-loxP-mmp14b-P2A-tagBFP); Tg(myl7:CreERT2)* zebrafish were incubated at 39 °C for one hour daily to induce *mmp14b* overexpression.

### Immunostaining, confocal imaging, and quantification

Adult zebrafish hearts were extracted and fixed overnight in 4% (vol/vol) paraformaldehyde (PFA) at 4 °C, washed with 1× PBS, and cryopreserved in 30% (wt/vol) sucrose before embedding in O.C.T. (Tissue-Tek) and stored at −80 °C. 12 μm sections were collected for immunostaining analysis. For PCNA/Mef2 immunostaining, sections were washed twice with PBST (1× PBS, 0.1% Triton X-100), twice with dH_2_0, followed by permeabilization in 3% (vol/vol) H_2_O_2_ in methanol for 1 h at room temperature. Sections were washed twice in dH_2_0, twice in PBST, and then incubated in blocking solution (1× PBS, 2% (vol/vol) sheep serum, 0.2% Triton X-100, 1% DMSO) for 30 min to 1 h. Primary antibodies were incubated at 37 °C for 3 h, followed by three washes in PBST and incubation with secondary antibodies at room temperature for at least 1 h. Sections were washed in PBST and mounted in Vectashield Antifade mounting medium (Vector Labs). For collagen hybridization peptide (CHP), GFP, DsRed (mScarlet), RFP, mCherry, Myosin Heavy Chain (MHC), Col12a1a, Tnfa, Cxcr4b, Vcl, and F-actin immunostaining, sections were washed twice with PBST, followed by permeabilization in PBST× (1× PBS, 0.5% Triton X-100) for 0.5–1 h at room temperature, and incubated in blocking solution and then stained with primary antibodies in blocking solution (or CHP, heat dissociated at 80 °C for 5 min prior to dilution in blocking solution according to manufacturer’s instructions) overnight at 4 °C. Washes and incubation with secondary antibodies were performed as above. For Aldh1a2 immunostaining, sections were washed twice with PBST and permeabilization was performed by incubation in acetone for 15 min at −20 °C. For N2.261 immunostaining, sections were post-fixed with 4% PFA for 10 min, followed by antigen retrieval with sodium citrate (pH 6.0) for 20 min before blocking and staining. Sections were then incubated in blocking solution and stained with primary antibodies in blocking solution overnight at 4 °C. Washes and incubation with secondary antibodies were performed as above. Primary antibodies used in this study include: anti-PCNA (Abcam ab29) at 1:200, anti-Mef2 (Boster DZ01398-1) at 1:200, anti-GFP (Aves Labs GFP-1020) at 1:500, anti-DsRed (Takara 632496) at 1:300, anti-RFP (Invitrogen MA5-15257) at 1:500, anti-mCherry (Thermo Fisher M11217) at 1:200, anti-MYH1 (MHC, DSHB A4.1025) at 1:100, anti-Col12a1a (Boster DZ41260) at 1:200, anti-Aldh1a2 (Genetex GTX124302) at 1:100, collagen hybridizing peptide (CHP, 3Helix B-CHP) at 20 μM, anti-Tnfa (Abcam ab1793) at 1:100, anti-Cxcr4b (Abcam ab229623) at 1:500, anti-Vcl (Sigma-Aldrich V9131) at 1:250, anti-embCMHC (DSHB N2.261) at 1:25, and Alexa Fluor 647-Phalloidin (Thermo Fisher A22287) at 1:500. Alexa Fluor-coupled secondary antibodies were used (Thermo Fisher) at 1:500.

Imaging of immunostained sections was performed using a Zeiss LSM800 Observer confocal microscope with a 25× LD LCI Plan-Apochromat objective or a Leica Mica confocal microscope with 20× HC PL FLUOTAR and 63× HC PL APO objectives. Quantification of cardiomyocyte proliferation was performed in the area 100 μm proximal from the wound border in three nonconsecutive sections exhibiting the largest injured area from each heart (PCNA+Mef2+ CMs/total Mef2+ CMs). Quantification of CHP intensity was performed in the injured area 100 μm distal from the wound border in three nonconsecutive sections exhibiting the largest area from each heart. Quantification of Aldh1a2 intensity and % area was performed in the entire injury area in three nonconsecutive sections exhibiting the largest injured area from each heart. Quantification of cortical CM coverage in CM:*mmp14b* OE ventricles was performed by measuring the length of MHC+ signal along the apex of the wound and presented as the percentage of the length of the wound apex. Quantification of macrophage aspect ratio was performed in *mpeg1*:EGFP+ cells from thin, fixed cardiac tissue in the area 50 μm distal and 50 μm proximal to border zone CMs (BZ) or within the injured area (excluding the epicardium) in three nonconsecutive sections exhibiting the largest area from each heart. GFP signal was thresholded using the default algorithm and a binary image was used to quantify aspect ratio. All quantifications described above were performed in ImageJ (v2.9.0).

To visualize cardiomyocyte protrusions at the border zone, wild-type hearts were fixed overnight in 4% PFA at 4 °C and incubated in 30% sucrose/PBS overnight before embedding in Tissue-Tek OCT medium. 60 μm thick cryosections were obtained and allowed to dry at room temperature (RT) for 3 h. Sections were then washed with 1× PBS twice at RT to remove OCT, permeabilized in 1× PBST (0.5% Triton X-100) for 2 h, and incubated overnight with Alexa Fluor 647-Phalloidin (Thermo Fisher A22287) in blocking solution (1× PBS, 2% (vol/vol) sheep serum, 0.2% Triton X-100, 1% DMSO). Sections were washed 3× in PBST (0.1% Triton X-100) and embedded in Vectashield Antifade mounting media (Vector Labs). Thick sections were imaged using a Zeiss LSM800 Observer confocal microscope with a 25× LD LCI Plan-Apochromat objective or a Leica Mica confocal microscope with a 20× HC PL FLUOTAR objective. Cardiomyocyte protrusions were quantified using a maximum intensity projection image from at least 2 sections exhibiting the largest injured area and containing largely trabecular cardiomyocytes. Quantification of protrusions into the injured area and measurement of length were performed in ImageJ (v2.9.0).

### Histological staining, imaging, and quantification

Adult zebrafish hearts collected for histology at 10 dpci were extracted and fixed overnight in 10% formalin at 4 °C, washed with PBS, dehydrated in ethanol gradient, washed in Xylene followed by 50% Xylene/ 50% paraffin, and embedded in paraffin. 5 μm sections were obtained for histological analysis. Adult zebrafish hearts collected for histology at 21, 30, and 60 dpci were extracted and fixed overnight in 4% paraformaldehyde at 4 °C, washed with PBS, cryopreserved in 30% sucrose and embedded in O.C.T. (Tissue-Tek) and stored at −20 °C. 12 μm sections were obtained for histological analysis.

Picrosirius Red staining was performed using the Picrosirius Red stain (Morphisto). FFPE sections were incubated in Bouin’s solution overnight at room temperature, followed by a rinse in running water for 10 min. Incubation with Picrosirius Red solution and subsequent steps were performed according to manufacturer’s instructions. Cryosections were post-fixed 2 min in 4% paraformaldehyde at room temperature, followed by overnight incubation in Bouin’s solution at room temperature and washed in running water for 10 min. Sections were incubated in Picrosirius Red solution for 90 min at room temperature and subsequent steps were performed according to manufacturer’s instructions. Sections were mounted in Entellan (Sigma).

AFOG staining was performed using the Acid Fuchsin Orange G Kit (Biognost). FFPE sections were incubated in Bouin’s solution overnight at room temperature, followed by a rinse in running water for 10 min. Subsequent steps were performed according to manufacturer’s instructions. Sections were mounted in Entellan (Sigma).

Imaging of histological staining was performed with the Leica Mica using widefield imaging, with a 10× or 20× HC PL FLUOTAR objectives. Scar area was quantified from Picrosirius Red staining of three to five nonconsecutive sections from each heart relative to the ventricle area in ImageJ (v2.9.0).

### In situ hybridization

In situ hybridization from ventricle sections was performed on cryosections according to standard procedures. Briefly, sections were dried at 50 °C, fixed in 4% (vol/vol) paraformaldehyde, treated with 5 μg/mL Proteinase K (Roche) and post-fixed in 4% (vol/vol) paraformaldehyde. Acetylation of sections was performed by incubation in 0.1 M triethanolamine/0.25% acetic anhydride with stirring, followed by washing in PBS, and prehybridization at 70 °C in hybridization buffer (50% formamide, 5× SSC, 0.1% Tween-20, 50 μg/mL heparin, 500 μg/mL yeast tRNA, pH 6.0). Digoxigenin-labeled RNA probes (200 ng/mL) were hybridized to tissue sections overnight at 70 °C in a hybridization oven. Washes with 2× SSC and 0.2× SSC were performed at 70 °C, followed by washes in PBT (1× PBS, 2 mg/mL BSA, 0.1% Triton X-100) and blocking in PBT/10% inactivated sheep serum. Sections were incubated overnight at 4 °C with Anti-Digoxigenin-AP antibody (1:2000, Roche) in blocking solution. Sections were washed in PBT and alkaline phosphatase buffer and stained with BM Purple (Roche) staining solution. Primers (Supplementary Table 1) were used to amplify PCR products, which were then used to generate in situ hybridization probes with T7 RNA polymerase (Promega) and Digoxigenin-UTP (DIG RNA Labeling Mix, Roche). Imaging of in situ hybridized sections was performed using a Nikon SMZ25 stereomicroscope.

### Hybridization chain reaction staining

mRNA expression was verified by HCR^TM^ RNA fluorescence in situ hybridization (RNA-FISH). Ventricular tissue was extracted and treated with 20 μg/mL Heparin for 5 min to remove erythrocytes before fixing in 4% PFA at room temperature for 1 h, cryopreserving in 30% sucrose overnight, and embedding in O.C.T. For *mmp14b*, *vwf*, *postnb* and *col12a1a* detection, antigen retrieval was performed, followed by permeabilization in 3% H_2_O_2_ in methanol for 1 h at room temperature before proceeding with RNA-FISH following the manufacturer’s protocol (Molecular Instruments). For *mrc1b* detection, sections were post-fixed with 4% PFA for 10 min at 4 °C, treated with 10 μg/mL Proteinase K for 10 min at 37 °C, and then refixed with 4% PFA for 10 min at room temperature before proceeding with RNA-FISH following the manufacturer’s protocol. Immunostaining was then performed according to the protocol above.

### DQ-Collagen staining

DQ-Collagen staining was performed according to Gamba et al.^60^ with the following alterations. Briefly, unfixed cryoinjured hearts were incubated overnight at 4 °C in 30% sucrose/PBS and embedded in OCT medium (Tissue-Tek). 12 μm cryosections were obtained and embedding medium was removed by washes in PBST (0.1% Triton X-100). Sections were preincubated for 5 min at RT in 1× reaction buffer (50 mM Tris-HCl, 150 mM NaCl, 5 mM CaCl_2_, 0.2 mM sodium azide, pH 7.6) and then incubated in 0.1 mg/mL DQ-Collagen (fluorescein conjugate, Thermo Fisher D12060) in 1× reaction buffer for 1 h at RT. Sections were washed 3× in 1× PBST (0.1% Triton X-100) and incubated in Alexa Fluor 647-coupled phalloidin in 1× PBST (0.1% Triton X-100) for 1 h at RT. Sections were washed 3× in 1× PBST (0.1% Triton X-100), mounted in Vectashield Antifade mounting medium (Vector Labs), and imaged with confocal microscopy as described above for immunostained sections.

### Live-imaging analysis of ventricular tissue slices

Zebrafish euthanasia and heart extraction were performed following previous reports^38^. After removal of blood and stopping the heartbeat, hearts were oriented with the former site of atrium facing up and mounted in 2.5% low EEO agarose (Sigma-Aldrich #A0576) dissolved in LAF medium [Leibovitz’s L15-Medium with GlutaMAX™ (Gibco #31415029)/1 × MEM Non-Essential Amino Acids Solution (Gibco #11140035)/20 mM 2,3-Butanedione monoxime (Sigma-Aldrich #B0753)/10% Fetal Bovine Serum (Gibco #A3160401)/100 μg/ml Primocin (InvivoGen #ant-pm-1)/1% Pen/Strep (Gibco #15070063)] in a cryomold. After solidifying for 5 min, agarose blocks were sectioned using a vibratome [Precisionary Instruments #VF-310-0Z, slice thickness = 100 μm, speed = 1.8, oscillation frequency = 5] and collected in LAF medium. Sections were transferred to μ-Slide 8-Well High ibiTreat plate (ibidi #80806) precoated with 11 μg/cm^2^ fibronectin (Sigma-Aldrich #F0895) containing LAF medium. The medium was largely removed to allow sections to adhere to the plate during incubation at 28.5 °C for 90 min. LAF medium was then supplied back to the plate storing at 28.5 °C before imaging.

The ibiTreat 8-well plate was directly used for time-lapse imaging using a Nikon AX confocal microscope. For Tg(*myl7*:*LIFEACT-EGFP; myl7:nls-DsRed*) and Tg(*myl7*:*actn3b-EGFP; myl7:nls-DsRed*), imaging was performed with a 1.5 μm *z*-step size and a 15-min interval using a 20× objective for 12 h. For *Tg(myl7*:*lck-mScarlet; mpeg1:EGFP*), imaging was performed with a 1.5 μm *z*-step size and a 15-min interval using a 20× objective for 6 h. Time-lapse videos were corrected for drift using the StackReg (v2.0.0) plug-in in ImageJ (v2.9.0). Cardiomyocyte protrusion ends were tracked manually, and migratory parameters were measured using the TrackMate (v7.10.2) plug-in^100^ in ImageJ (v2.9.0).

### Macrophage ablation

Ventricles from *Tg(mpeg:NTR-YFP)* adult fish were cryoinjured as described above. Cryoinjured fish were then incubated in a 5 μm nifurpirinol or DMSO-control water bath from 4 to 6 days following cryoinjury (replenished daily). Hearts were extracted at 7 dpci and subjected to immunostaining with GFP antibody to check macrophage ablation efficiency and phalloidin staining to quantify CM protrusion at the border zone as described above.

To ablate resident macrophages, *Tg(mpeg1:EGFP)* adult fish were IP injected with 10 μl clodronate liposomes (5 mg/ml) (Liposoma, Amsterdam, The Netherlands) or PBS 8 days prior to cryoinjury. Hearts were extracted at 7 or 10 dpci followed by cryosection, CHP staining, and phalloidin staining, as described above. Quantification of CHP intensity and CM protrusion at the border zone were performed as described above.

To ablate macrophages at later timepoints, *Tg(mpeg1:EGFP)* adult fish were IP injected with 10 μl clodronate liposomes (5 mg/ml) (Liposoma, Amsterdam, The Netherlands) or PBS control liposomes at 3, 6, and 9 dpci before collection of hearts at 10 dpci. To study the impact of macrophage ablation on *mmp14b* overexpression in CMs, *Tg(hsp70l:loxP-EGFP-loxP-mmp14b-P2A-tagBFP); Tg(−5.1myl7:CreERT2)* adult fish were IP injected with clodronate liposomes or PBS liposomes 1 day prior to cryoinjury and every 4 days afterwards. Tamoxifen/ethanol injections and heat shock were performed to induce *mmp14b* overexpression as described above. Hearts were extracted at 10 or 21 dpci followed by cryosection and phalloidin or MHC staining as described above. Quantification of CM protrusion at the border zone and CM cortical coverage were performed as described above.

### Single-cell RNA-sequencing of border zone cells

Zebrafish hearts were cryoinjured as above and extracted at 7 dpci. The border zone was roughly dissected and a pool of border zone cells from 8 hearts was dissociated using the Pierce Primary Cardiomyocyte Isolation kit (Thermo Fisher 88281) according to manufacturer instructions. The cell suspensions were depleted for dead cells using a LeviCell 1.0 device (Levitas Bio) and were counted with a Moxi cell counter and diluted according to manufacturer’s protocol to obtain 10,000 single cell data points per sample. Each sample was run separately on a lane in Chromium controller with Chromium Next GEM Single Cell 3ʹ Reagent Kits v3.1 (10× Genomics). Single-cell RNA-seq library preparation was done using standard protocols. Sequencing was done on a Nextseq2000 and raw reads were aligned against the zebrafish genome (DanRer11) and counted by StarSolo^101^, followed by secondary analysis in Annotated Data Format. Preprocessed counts were further analyzed using Scanpy (v1.9)^102^. Basic cell quality control was conducted by taking the number of detected genes and mitochondrial content into consideration. We removed 23 cells in total that did not express more than 300 genes or had a mitochondrial content greater than 40%. Furthermore, we filtered 9874 genes if they were detected in less than 30 cells (<0.01%). Raw counts per cell were normalized to the median count over all cells and transformed into log space to stabilize variance. We initially reduced dimensionality of the dataset using PCA, retaining 50 principal components. Subsequent steps, like low-dimensional UMAP embedding (McInnes & Healy, https://arxiv.org/abs/1802.03426) and cell clustering via community detection (Traag et al., https://arxiv.org/abs/1810.08473), were based on the initial PCA. Final data visualization was done by Scanpy and CellxGene packages (DOI 10.5281/zenodo.3235020). Differential gene expression analysis (DEG) was performed using Scanpy. Gene ontology analysis of DEGs was performed using Panther (v18.0)^103^ and visualized using REVIGO (v1.8.1)^104^.

### Gene expression analysis by RT-qPCR

RNA from adult zebrafish ventricles and larvae was isolated by resuspension of cells in TRIzol (Thermo Fisher), addition of chloroform, and centrifugation according to manufacturer’s instructions. RNA from the aqueous layer was purified using the RNA Clean and Concentrator Kit (Zymo). Total RNA was reverse transcribed to cDNA using the iScript Advanced cDNA kit (Biorad) according to manufacturer’s instructions. qPCR was performed using SsoAdvanced Universal SYBR Green Supermix (Biorad) and the LightCycler 480II (Roche). Primers were used for gene expression analysis by RT-qPCR can be found in Supplementary Table 1.

### Sorting of cells by FACS for gene expression analysis

*Tg(myl7:mKATE-CAAX); Tg(mpeg1:EGFP)* adult fish were cryoinjured and ventricles extracted at 7 dpci (pools of 3 ventricles per biological replicate). Tissue was dissociated using the Pierce Primary Cardiomyocyte Isolation kit (Thermo Fisher 88281) according to manufacturer’s instructions and single cells were resuspended in DMEM/10% FBS/1× glutamate. mKATE+ CMs, EGFP+ macrophages, and mKATE-EGFP- cells were sorted using a FACS Aria III and subjected to gene expression analyses as above.

### Isolation of CMs from CM:*A-Fos* for gene expression analysis

6 ventricles from control or CM:*A-Fos* fish at 7 dpci were isolated per biological replicate. Tissue was dissociated using the Neonatal Heart Dissociation kit (Miltenyi 130-098-373) according to manufacturer’s instructions, with the following changes: hearts were not minced prior to incubation with enzyme and the 37C_mr_NHDK_1 gentleMACS octo-dissociator protocol was run twice, but only for the first 30 min to remove the last mechanical disruption step. Cardiomyocytes were isolated by sucrose density gradient centrifugation according to previously published protocols^43^. Gene expression analysis by RT-qPCR was performed as described above and primers used for gene expression analysis can be found in Supplementary Table 1.

### Statistics and reproducibility

All statistical analyses for experimental data were performed in GraphPad Prism 10. Distribution of data from all sample groups was assessed using the Shapiro-Wilks normality test. Comparative statistics between two sample groups was performed using the unpaired two-sided *t*-test for parametric data or the two-sided Mann–Whitney test for nonparametric data. Comparative statistics between more than two sample groups was performed using ordinary one-way ANOVA for parametric data or the Kruskal–Wallis test for nonparametric data. Specific multiple comparison tests used for experimental data with more than two sample groups can be found in the figure legends.

For immunostaining and histological staining, the images presented were representative of the following number of independent ventricles: Fig. 1D (*n* = 4 ventricles), Fig. 1E (*n* = 5 ventricles), Fig. 1F (*n* = 5 ventricles), Fig. 3A (*n* = 3 ventricles), Fig. 3G (*n* = 5 ventricles), Fig. 6F (Control *n* = 3 ventricles, CM:*AFos* *n* = 3 ventricles), Fig. 7A (*n* = 5 ventricles), Fig. 7B (*n* = 4 ventricles), Fig. 7C (*vwf* *n* = 4 ventricles, *mpeg* *n* = 4 ventricles, *postnb* *n* = 3 ventricles, *col12a1a* *n* = 7 ventricles, MHC *n* = 7 ventricles), and Fig. 8F (*n* = 5 wild-type ventricles, *n* = 6 *mmp14b* mutant ventricles).

### Reporting summary

Further information on research design is available in the Nature Portfolio Reporting Summary linked to this article.

## Supplementary information

Supplementary Information

Peer Review file

Description of Additional Supplementary Files

Supplementary Movie 1

Supplementary Movie 2

Supplementary Movie 3

Supplementary Movie 4

Supplementary Movie 5

Supplementary Movie 6

Supplementary Movie 7

Reporting Summary

## Source data

Source Data

## Data Availability

Single-cell RNA-seq data from this study have been deposited in the Gene Expression Omnibus (GEO) database under the accession number GSE251856. Published ATAC-seq data from regenerating *gata4*:EGFP+ cardiomyocytes were accessed from GSE130940. Source data are provided with this paper.
