# Supplementary material for: Border-zone cardiomyocytes and macrophages regulate extracellular matrix remodeling to promote cardiomyocyte protrusion during cardiac regeneration
Source: Nat Commun. 2025 Apr 23;16:3823. doi: 10.1038/s41467-025-59169-4 (PMC12019606; doi:10.1038/s41467-025-59169-4)
Supplement: Supplementary file 1 — Supplementary Information [file 41467_2025_59169_MOESM1_ESM.pdf]

Border-zone cardiomyocytes and macrophages regulate extracellular matrix remodeling to promote cardiomyocyte protrusion during cardiac regeneration

Supplementary Information Contents

Supplementary Figure 1: Characterizing cardiomyocyte protrusions at the border zone

Supplementary Figure 2: Time-lapse imaging and tracking of trabecular CM protrusions

Supplementary Figure 3: Characterizing macrophages at the wound border zone

Supplementary Figure 4: Absence of macrophages results in defects in scar composition, CM protrusion and organismal survival

Supplementary Figure 5: Quality control and initial analyses of scRNA-seq from border zone cells at 7 dpci

Supplementary Figure 6: Gene ontology analysis of CMs in border zone scRNA-seq at 7 dpci

Supplementary Figure 7: Gene ontology analysis of immune cell clusters in border zone scRNA-seq at 7 dpci

Supplementary Figure 8: *mmp14b* expression in regenerating zebrafish hearts

Supplementary Figure 9: *mmp14b* mutant embryos display no genetic compensation from *mmp14a*

Supplementary Figure 10: Endocardial and fibroblast response in *mmp14b* mutants

Supplementary Figure 11: *mmp14b* OE in cardiomyocytes does not accelerate heart regeneration

Supplementary Table 1: PCR primers used in this study

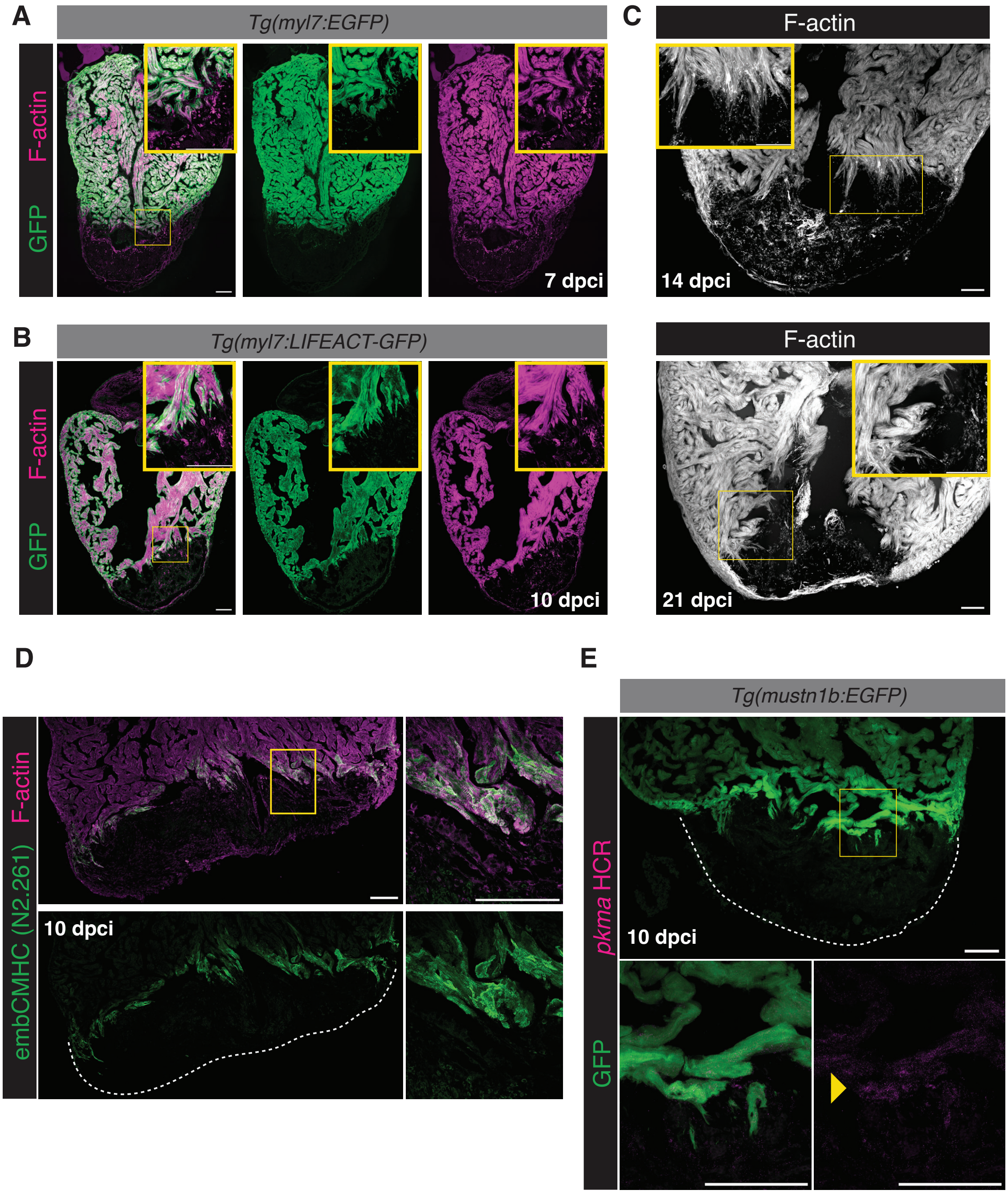

Supplementary Figure 1: Characterizing cardiomyocyte protrusions at the border zone.

(A) Phalloidin and GFP immunostaining in *Tg(myl7:EGFP)* ventricles at 7 dpci (n=6 ventricles). Yellow box denotes the zoomed image from the wound border zone.

(B) Phalloidin and GFP immunostaining in *Tg(myl7:LIFEACT-GFP)* ventricles at 10 dpci (n=5 ventricles). Yellow box denotes the zoomed image from the wound border zone.

(C) Phalloidin staining of F-actin in thick cryosections of zebrafish ventricles at 14 (n=8 ventricles) and 21 dpci (n=6 ventricles). Yellow box denotes the zoomed image from the wound border zone.

(D) Phalloidin (F-actin) and embCMHC (N2.261) staining at 10 dpci (n=3 ventricles). White dashed line indicates the apical wound and the yellow box denotes the zoomed image from the wound border zone.

(E) GFP immunostaining and *in situ* hybridization chain reaction (HCR) of *pkma* in *Tg(mustn1b:EGFP)* ventricles (n=4 ventricles) at 10 dpci. White dashed line indicates the apical wound and the yellow box denotes the zoomed image from the wound border zone. The yellow arrowhead points to *pkma* enrichment in border zone CMs.

Scale bars: 100 μm.

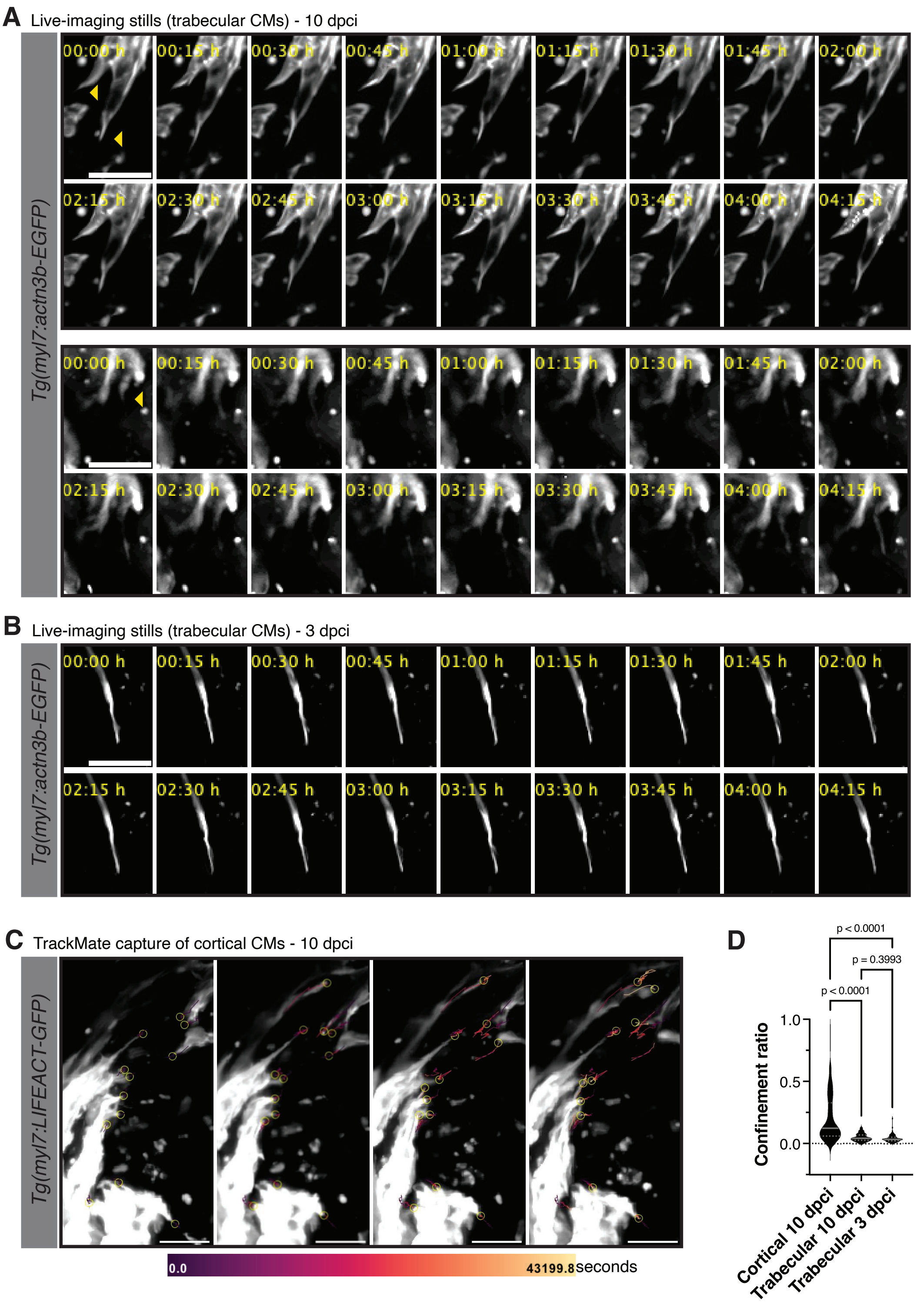

Supplementary Figure 2: Time-lapse imaging and tracking of trabecular CM protrusions.

(A) Time-lapse imaging of *Tg(myl7:actn3b-GFP)*+ trabecular CMs at 10 dpci. Yellow arrowheads point to CM protrusions that display a net positive migration into the injured area.

(B) Time-lapse imaging of *Tg(myl7:actn3b-GFP)*+ trabecular CMs at 3 dpci.

(C) TrackMate capture of *Tg(myl7:LIFEACT-GFP)*+ cortical CM protrusions at 10 dpci. Yellow circles denote the ends of CM protrusions that were tracked over a 12-hour time-lapse imaging period. Tracks were color-coded based on time.

(D) Quantification of confinement ratio in CM protrusions at the border zone in *Tg(myl7:LIFEACT-GFP)*+ cortical CMs at 10 dpci (n=114 protrusions from 6 ventricles) and *Tg(myl7:actn3b-EGFP)*+ trabecular CMs at 3 (n=76 protrusions from 5 ventricles) and 10 (n=84 protrusions from 4 ventricles) dpci. Data are presented as violin plots of all points with solid gray lines indicating the median and dotted gray lines indicating 25th and 75th percentile. P-values were calculated using a Kruskal-Wallis test with Dunnett's multiple comparisons test. Source data are presented in the Source Data file.

Scale bars: 20 μm.

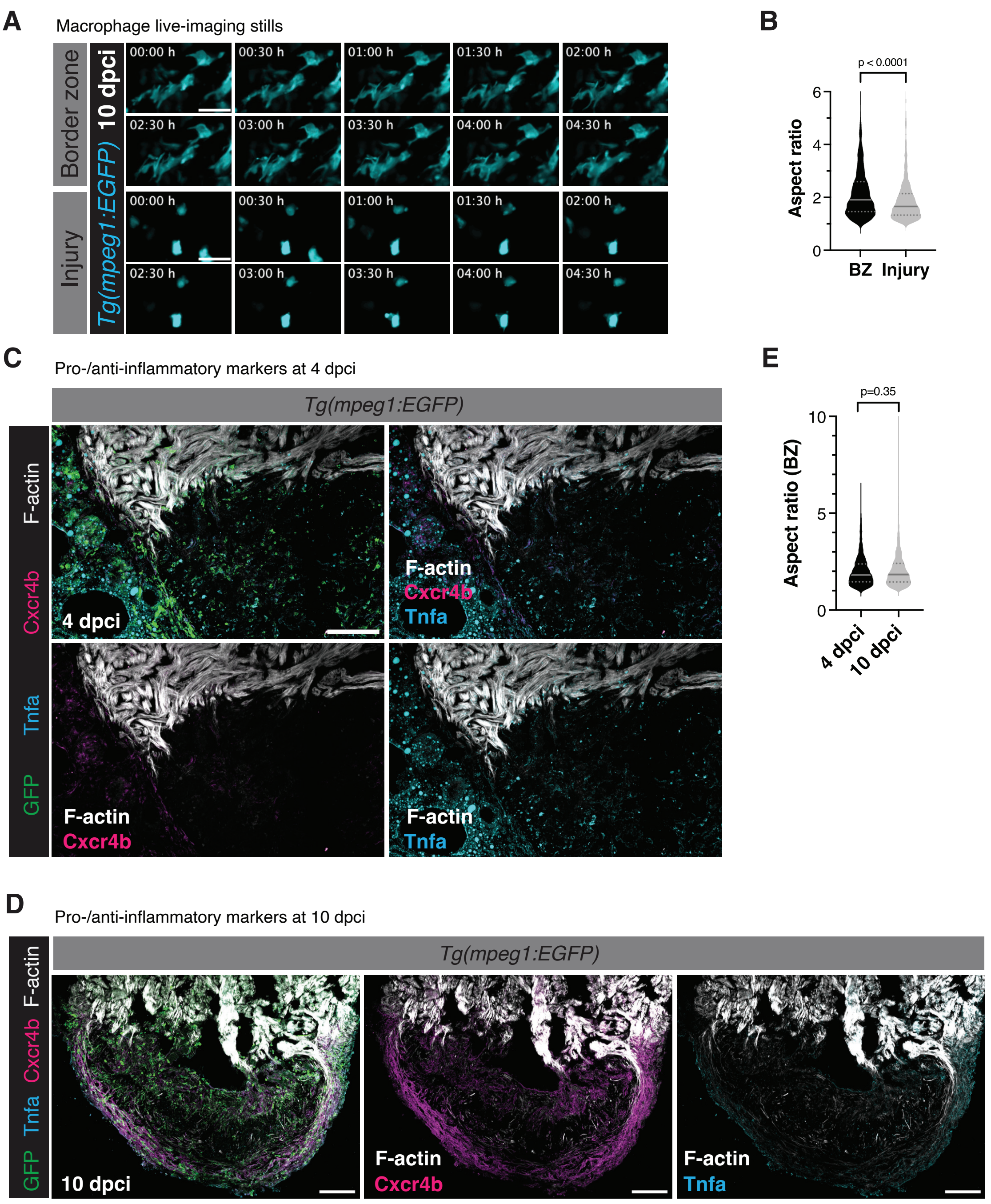

Supplementary Figure 3: Characterizing macrophages at the wound border zone.

(A) Time-lapse imaging of *Tg(myl7:lck-mScarlet); Tg(mpeg1:EGFP)* ventricular sections at the wound border zone at 10 dpci.

(B) Quantification of aspect ratio of *mpeg1*:EGFP+ cells 50 μm proximal and distal to the wound border (BZ, n=1235 macrophages in 4 ventricles) and within the injury (n=1267 macrophages in 4 ventricles) in *Tg(mpeg1:EGFP)* ventricles at 10 dpci. Data are presented as violin plots of all points with solid gray lines indicating the median and dotted gray lines indicating 25th and 75th percentile. P-values were calculated using a two-sided Mann-Whitney test. Source data are presented in the Source Data file.

(C) GFP, Tnfa, Cxcr4b, and F-actin immunostaining in *Tg(mpeg1:EGFP)* ventricles at 4 dpci (n=5 ventricles).

(D) GFP, Tnfa, Cxcr4b, and F-actin immunostaining in *Tg(mpeg1:EGFP)* ventricles at 10 dpci (n=7 ventricles).

(E) Quantification of aspect ratio of *mpeg1:*EGFP+ cells 50 μm proximal and distal to the wound border (BZ) in *Tg(mpeg1:EGFP)* ventricles at 4 (n=1205 macrophages in 4 ventricles) and 10 (n=1235 macrophages in 4 ventricles) dpci. Data are presented as violin plots of all points with solid gray lines indicating the median and dotted gray lines indicating 25th and 75th percentile. P-values were calculated using a two-sided Mann-Whitney test. Source data are presented in the Source Data file.

Scale bar: 10 μm in **(A)** and 100 μm in **(B)** and **(D)**.

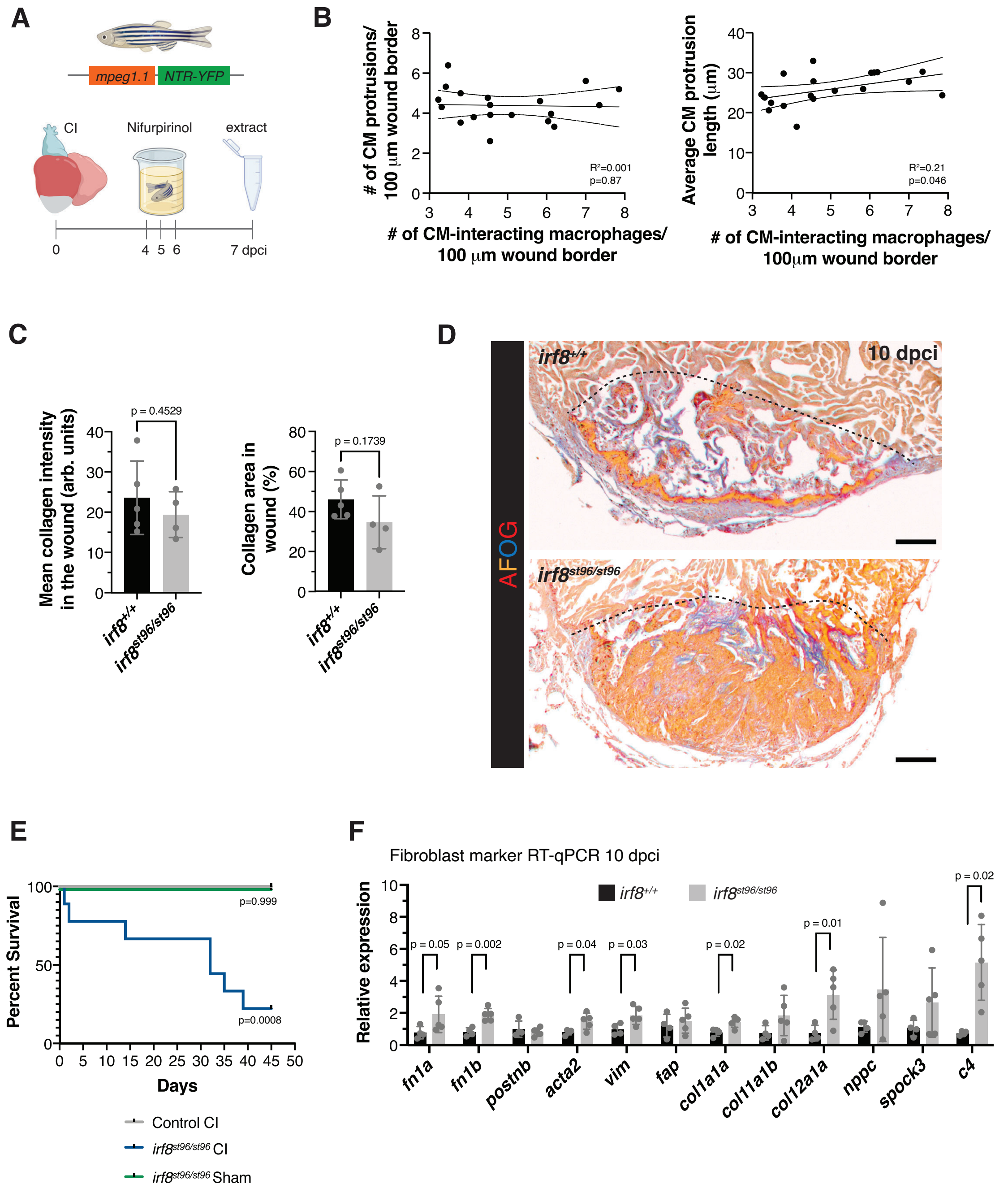

Supplementary Figure 4: Absence of macrophages results in defects in scar composition, CM protrusion and organismal survival.

(A) Schematic illustrating the macrophage ablation experimental set-up in *Tg(mpeg1.1:NTR-YFP)* ventricles. Created in BioRender. Beisaw, A. (2025) https://BioRender.com/4o9vrp6

(B) Correlation between the number of CM-interacting macrophages and the number and length of CM protrusions at 7 dpci (n=19 ventricles total) from *Tg(mpeg1.1:NTR-YFP)* zebrafish treated with DMSO (n=10 ventricles) and Nifurpirinol (n=9 ventricles). Dashed lines indicate 95% confidence intervals of the best-fit line and p-values were calculated using simple linear regression. Source data are presented in the Source Data file.

(C) Quantification of mean collagen intensity (arbitrary units, arb. units) and percentage of collagen area in the wound in $irf8^{st96/st96}$ mutant (n=4 ventricles) and wild-type sibling (n=5

ventricles) at 10 dpci. Data are presented as mean ±SD. P-value was calculated using an unpaired two-sided t-test. Source data are presented in the Source Data file.

(D) Acid fuchsin orange G (AFOG) staining in *irf8*$^{st96/st96}$ (n=5 ventricles) and wild-type sibling (n=5 ventricles) at 10 dpci. Black dashed lines indicate the approximate wound border.

(E) Kaplan-Meier survival curve of wild-type control (n=9 fish) and *irf8*$^{st96/st96}$ mutant fish after sham (n= 9 fish) or cryoinjury (n=9 fish). P-values compared to the control CI group were calculated using the Log-rank (Mantel-Cox) test. Source data are presented in the Source Data file.

(F) RT-qPCR analysis of fibroblast marker genes in *irf8*$^{st96/st96}$ mutant (n=5 ventricles) and wild-type sibling (n=4 ventricles) at 10 dpci. Data are presented as mean ±SD. P-values were calculated using an unpaired two-sided t-test or a two-sided Mann-Whitney test (*c4*). Source data are presented in the Source Data file.

Scale bars: 100 μm.

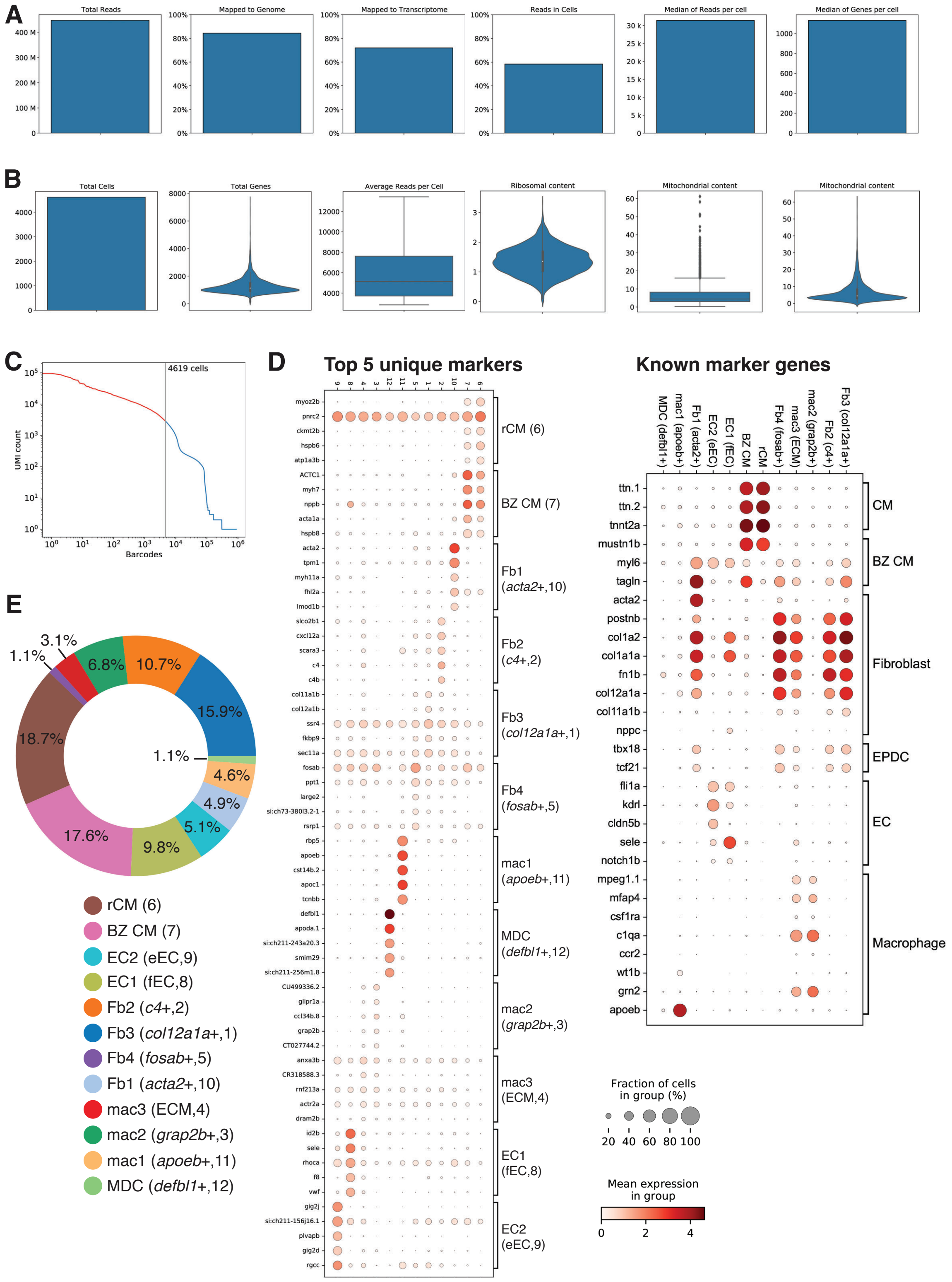

Supplementary Figure 5: Quality control and initial analyses of scRNA-seq from border zone cells at 7 dpci.

(A) (B) Quantitation and quality control metrics of scRNA-seq data, including total reads, mapped reads, median reads/genes per cell, ribosomal content, and mitochondrial content.

(C) Knee plot of scRNA-seq data displaying the cut-off used for further analysis.

(D) Dot plot of the top 5 unique marker genes for each cluster from the border zone scRNA-seq at 7 dpci (left). Dot plot of known marker genes from CMs, border zone CMs (BZ CMs), fibroblasts, epicardial-derived cells (EPDC), endothelial/endocardial cells (EC), and macrophages in the scRNA-seq data at 7 dpci (right). Red color denotes the mean expression within each group and size of the circle represents the fraction of cells expressing each gene within the group. MDC, myeloid-derived cells; Fb, fibroblast; mac, macrophage.

(E) Pie chart illustrating the fraction of cells within each cluster as a percentage of all cells.

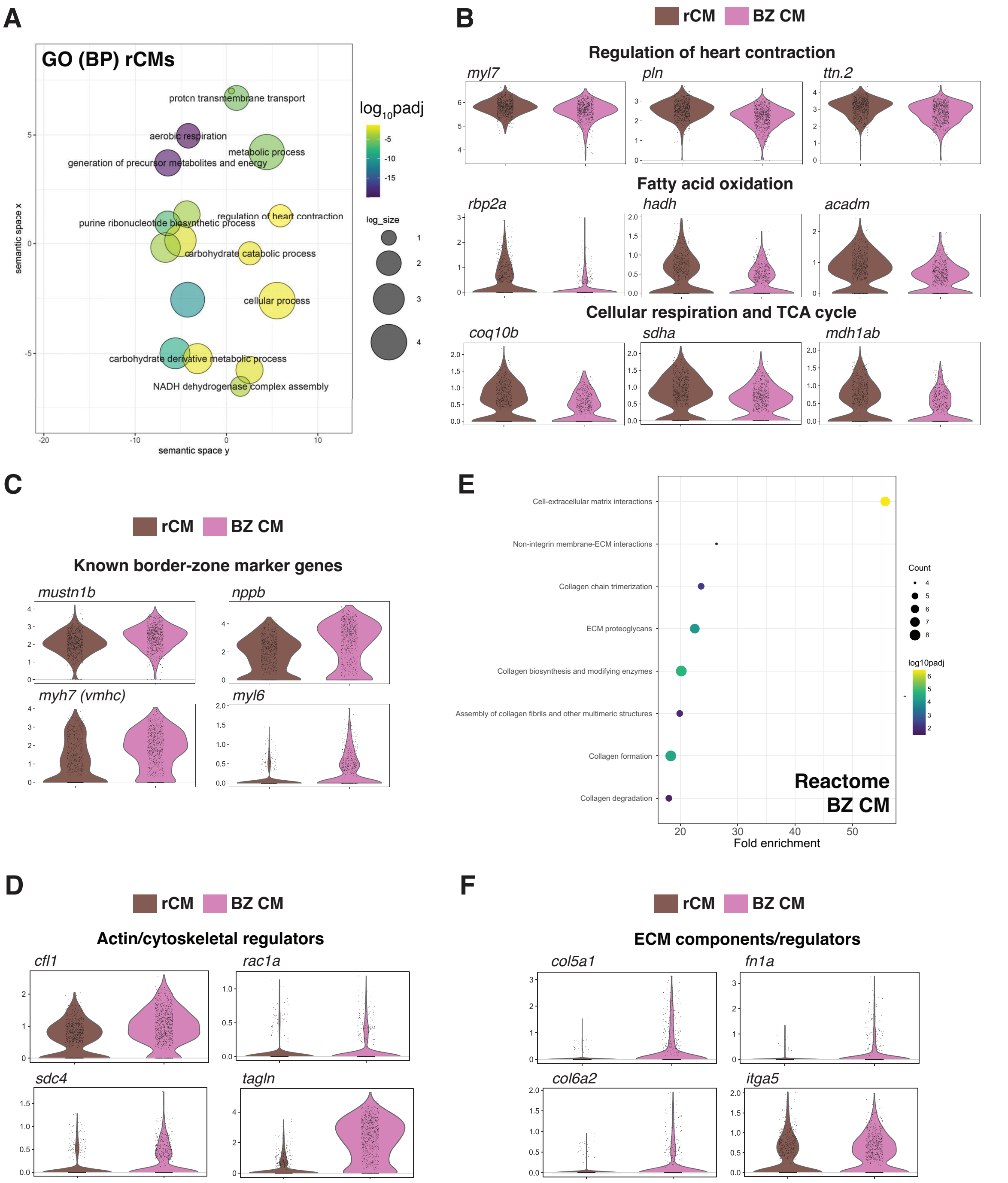

Supplementary Figure 6: Gene ontology analysis of CMs in border zone scRNA-seq at 7 dpci.

(A) Gene ontology (GO) analysis of biological processes (BP) in differentially expressed genes enriched in remote CMs compared to border zone CMs. log_{10}padj was calculated in comparison to all genes in the zebrafish genome using Fisher's exact test and corrected for multiple comparisons using the Bonferroni method. log_size corresponds to the log_{10}(number of annotations for the GO Term ID in zebrafish from the EBI GOA Database).

(B) Violin plots of expression of genes involved in regulation of heart contraction, fatty acid oxidation, and cellular respiration and TCA cycle in rCMs vs. BZ CMs clusters from scRNA-seq at 7 dpci.

(C) Violin plots of expression of known border-zone CM marker in rCMs vs. BZ CMs clusters from scRNA-seq at 7 dpci.

(D) Violin plots of expression of genes involved in actin cytoskeleton organization in rCMs vs. BZ CMs clusters from scRNA-seq at 7 dpci.

(E) Reactome pathway enrichment in genes upregulated in border zone CMs compared to remote CMs. Count refers to the number of genes included in each Reactome pathway term. $\log_{10}$padj was calculated in comparison to all genes in the zebrafish genome using Fisher's exact test and corrected for multiple comparisons using the Bonferroni method.

(F) Violin plots of expression of genes involved in ECM regulation in rCMs vs. BZ CMs clusters from scRNA-seq at 7 dpci.

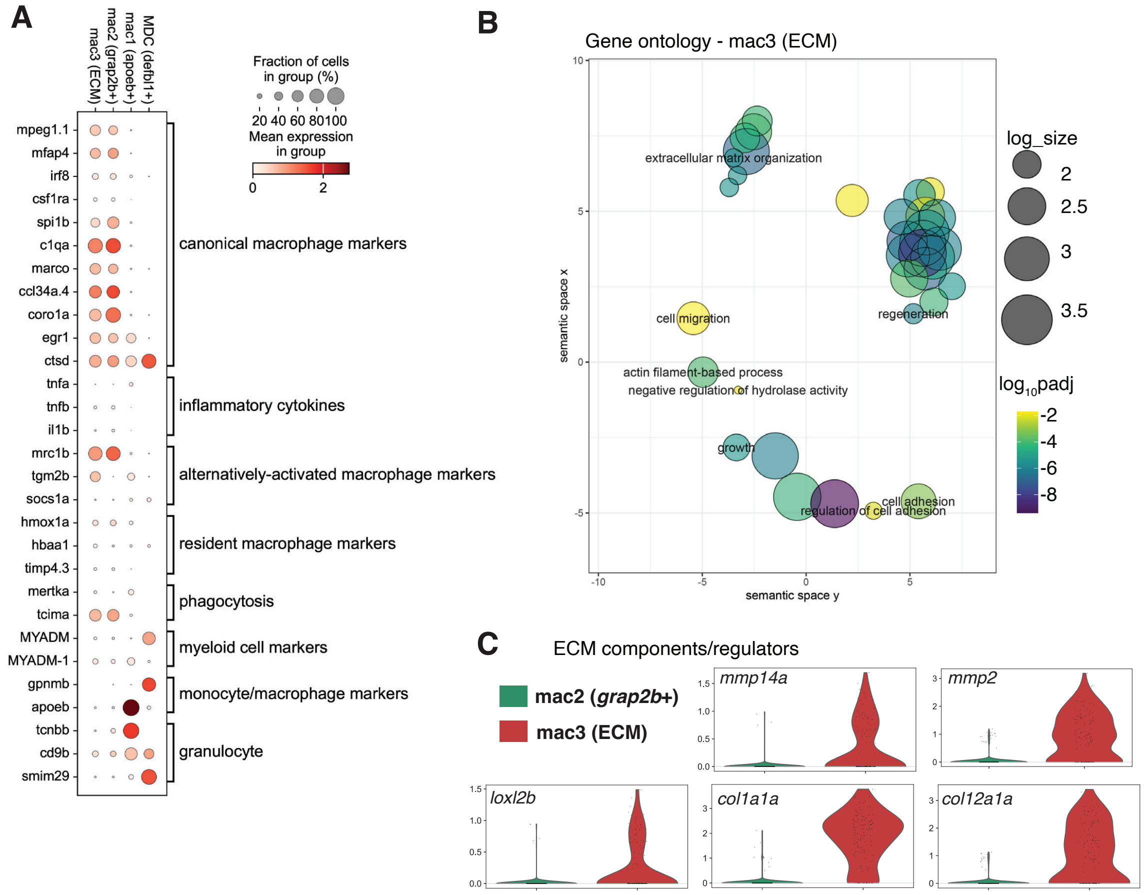

Supplementary Figure 7: Gene ontology analysis of immune cell clusters in border zone scRNA-seq at 7 dpci.

(A) Dot plot of monocyte/macrophage, inflammatory cytokines, phagocytosis, myeloid, and granulocyte marker genes from immune clusters in the scRNA-seq data at 7 dpci. Red color denotes the mean expression within each group and size of the circle represents the fraction of cells expressing each gene within the group.

(B) Gene ontology (GO) analysis of biological processes (BP) in differentially expressed genes enriched in mac2 compared to mac3(ECM). $\log_{10}$padj was calculated in comparison to all genes in the zebrafish genome using Fisher's exact test and corrected for multiple comparisons using the Bonferroni method. log_size corresponds to the $\log_{10}$(number of annotations for the GO Term ID in zebrafish from the EBI GOA Database).

(C) Violin plots of expression of genes involved in ECM regulation in mac3 (ECM) vs. mac2 clusters from scRNA-seq at 7 dpci.

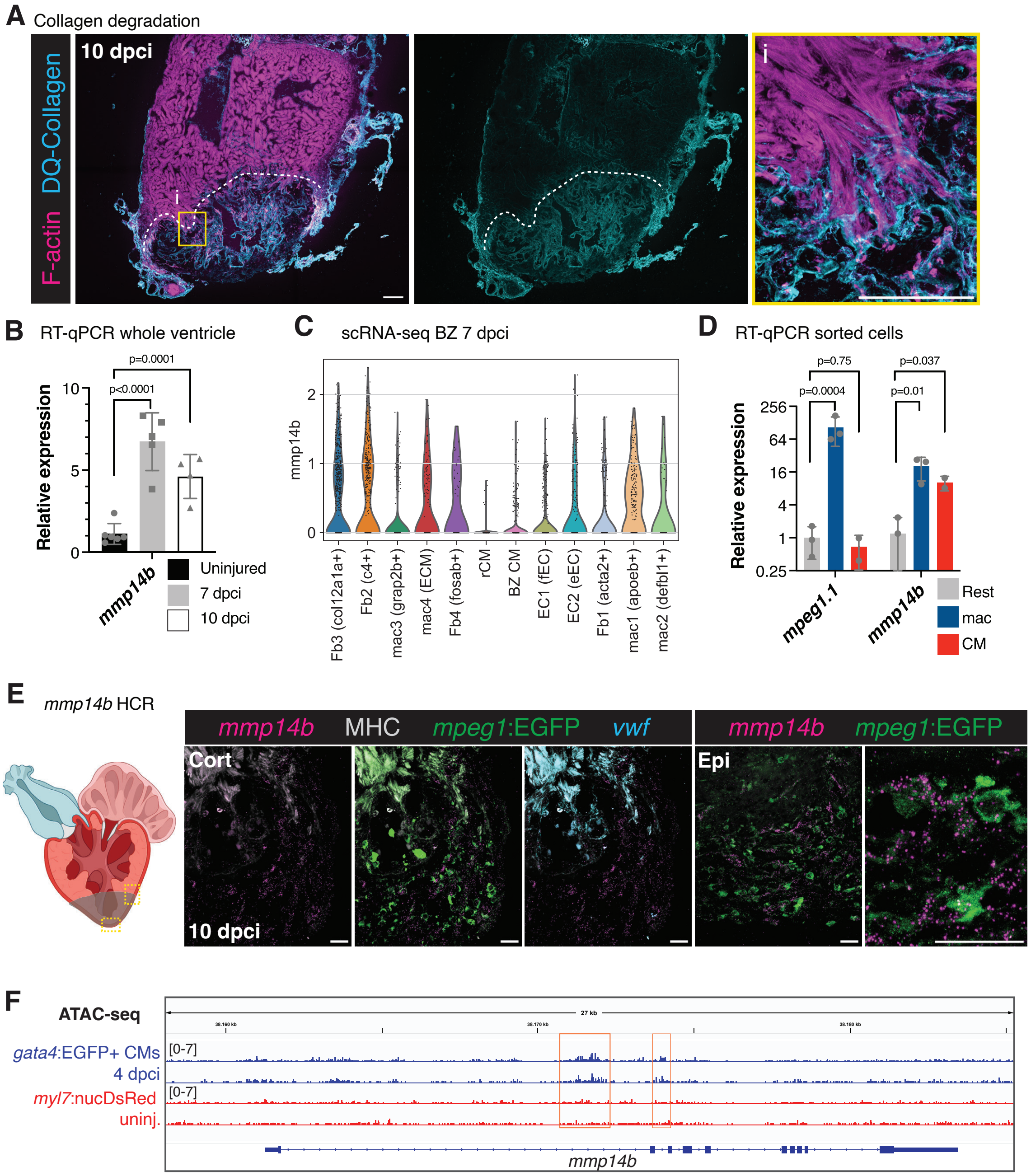

Supplementary Figure 8: *mmp14b* expression in regenerating zebrafish hearts.

(A) Incubation of fresh frozen sections with DQCollagen to fluorescently label collagenase activity in combination with phalloidin staining of F-actin in wild-type (n=6 ventricles) at 10 dpci. Yellow box denotes the zoomed image (i). White dashed lines indicate the approximate wound border.

(B) RT-qPCR analysis of *mmp14b* expression in whole ventricles at 7 (n=5 ventricles) and 10 (n=4 ventricles) dpci. Data are presented as mean ±SD. P-values were calculated using one-way ANOVA and Dunnett's multiple comparisons test. Source data are presented in the Source Data file.

(C) Violin plots of *mmp14b* expression in cell clusters from the border zone scRNA-seq at 7 dpci.

(D) RT-qPCR analysis of *mmp14b* expression in isolated CMs (n=2 independent pools of isolated CMs), macrophages (n= 3 independent pools of isolated macrophages), and non-

CM/non-mac (n=3 pools of isolated non-CM/non-mac) sorted by fluorescent activated cell sorting (FACS) from *Tg(mpeg1:EGFP; myl7:mKATE-CAAX)* ventricles at 7 dpci. Data are presented as mean ±SD. P-values were calculated using one-way ANOVA and Dunnett's multiple comparisons test. Source data are presented in the Source Data file.

(E) *mmp14b* and *vwf in situ* hybridization chain reaction (HCR) and GFP/MHC immunostaining in *Tg(mpeg1:EGFP)* ventricles, marking endocardial cells (*vwf*), macrophages (GFP) and cardiomyoctes (MHC) at 10 dpci. Yellow boxes in the schematic of the heart mark the cortical CM region and epicardial region depicted in the zoomed images. Cort, cortical CMs; Epi, epicardium. Schematic of the heart created in BioRender. Beisaw, A. (2025) https://BioRender.com/zek72tx

(F) ATAC-seq tracks (Integrated Genome Viewer v2.8.9) from regenerating *gata4*:EGFP+ CMs at 4 dpci and uninjured *myl7*:nucDsRed+ CMs showing CM-specific chromatin accessibility at the *mmp14b* locus.

Scale bars: 100 μm in **(A)** and 20μm in **(E)**.

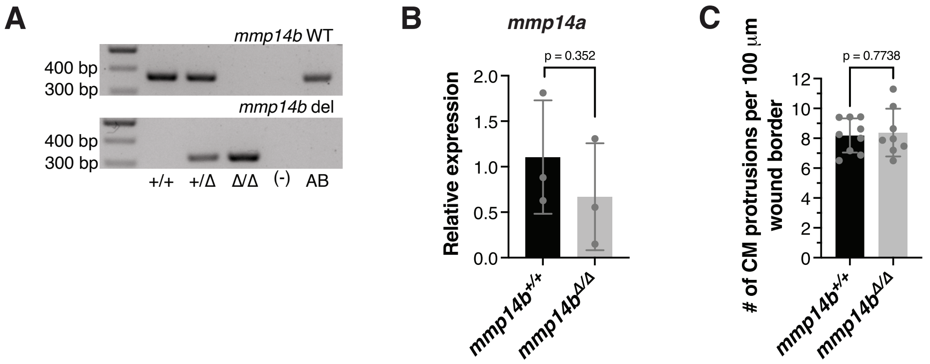

Supplementary Figure 9: *mmp14b* mutant embryos display no genetic compensation from *mmp14a.*

(A) Genotyping of genomic DNA from *mmp14b*$^{+/+}$, *mmp14b*$^{+/\Delta}$, and *mmp14b*$^{\Delta/\Delta}$ fish with primers specific for the *mmp14b* wild-type allele and *mmp14b* full locus deletion allele.

(B) RT-qPCR analysis of *mmp14a* expression from pools of *mmp14b*$^{+/+}$ and *mmp14b*$^{\Delta/\Delta}$ embryos at 72 hpf (n=3 pools of embryos per genotype). Data are presented as mean ±SD. P-values were calculated using an unpaired two-sided t-test. Source data are presented in the Source Data file.

(C) Quantification of the number of CM protrusions in *mmp14b*$^{\Delta/\Delta}$ mutant and wild-type sibling ventricles at 10 dpci. Data are presented as mean ±SD. P-values were calculated using an unpaired two-sided t-test. Source data are presented in the Source Data file.

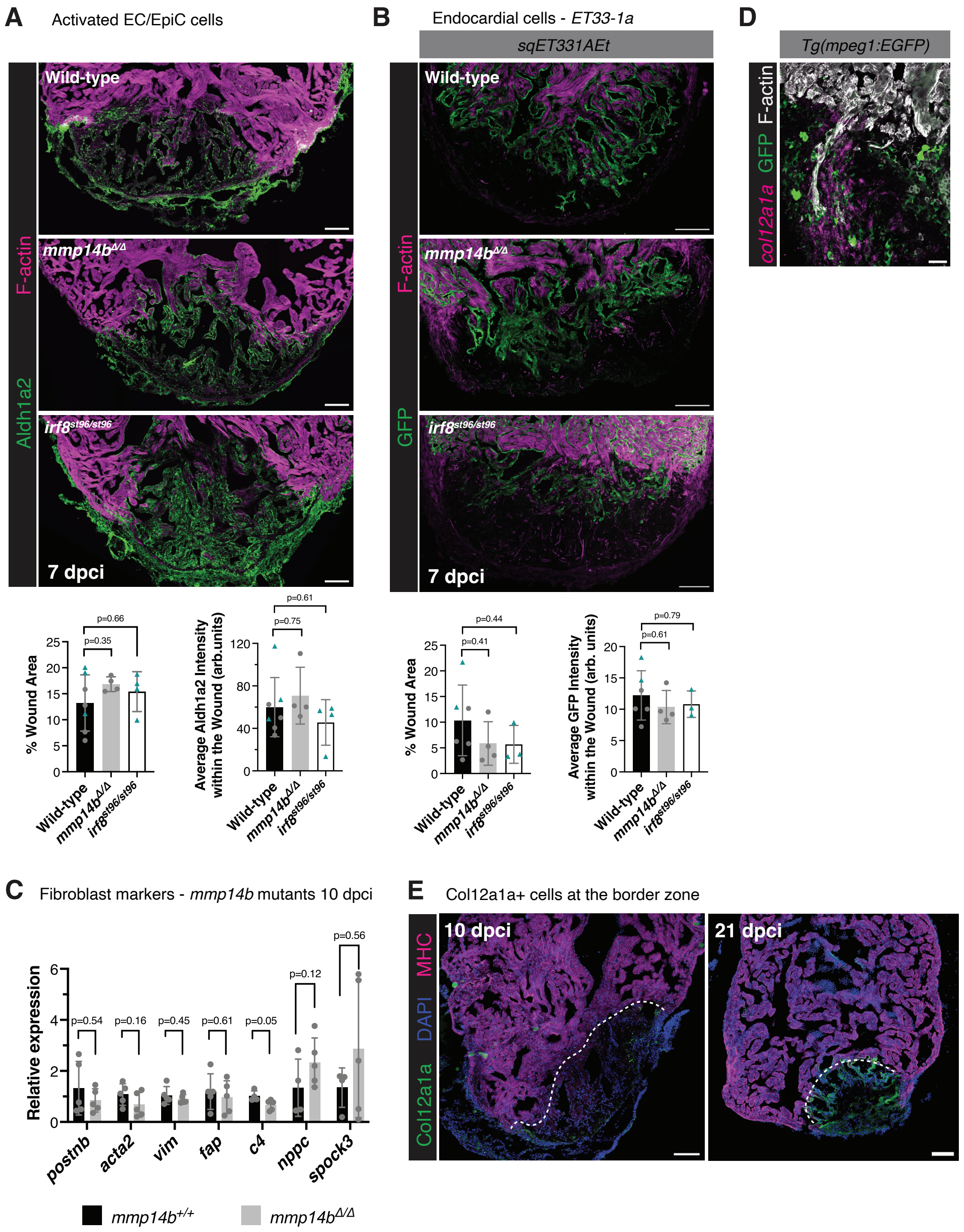

Supplementary Figure 10: Endocardial and fibroblast response in *mmp14b* mutants.

(A) Phalloidin and Aldh1a2 immunostaining in wild-type (n=7 ventricles), *mmp14b*$^{\Delta/\Delta}$ mutant (n=4 ventricles), and *irf8*$^{st96/st96}$ mutant (n=4 ventricles) at 7 dpci. Quantification of the percent Aldh1a2+ staining within the wound area and average Aldh1a2 intensity within the wound are shown on the bottom. Data are presented as mean ±SD. P-values were calculated using ordinary one-way ANOVA and Dunnett's multiple comparisons test. Wild-type siblings from *mmp14b* and *irf8* mutants were grouped together. Source data are presented in the Source Data file.

(B) Phalloidin and GFP immunostaining in wild-type (n=6 ventricles), *mmp14b*$^{\Delta/\Delta}$ mutant (n=4 ventricles), and *irf8*$^{st96/st96}$ mutant (n=3 ventricles) *sqet331aEt* (*ET33-1a*) at 7 dpci. Quantification of the percent GFP+ staining within the wound area and average GFP intensity within the wound are shown on the right. Data are presented as mean ±SD. P-values were calculated using ordinary one-way ANOVA and Dunnett's multiple comparisons test. Wild-type siblings from *mmp14b* and *irf8* mutants were grouped together. Source data are presented in the Source Data file.

(C) RT-qPCR analysis of fibroblast gene expression in *mmp14b*$^{\Delta/\Delta}$ mutant (n=5 ventricles) and wild-type sibling (n=5 ventricles) at 10 dpci. Data are presented as mean ±SD. P-values were calculated using an unpaired two-sided t-test or a two-sided Mann-Whitney test (*spock3*). Source data are presented in the Source Data file.

(D) *col12a1a* in situ hybridization chain reaction (HCR) and GFP/F-actin immunostaining in the cortical border zone in *Tg(mpeg1:EGFP)* at 10 dpci (n=5 ventricles).

(E) Col12a1a and Myosin heavy chain (MHC) immunostaining in 10 (n=4 ventricles) and 21 (n=7 ventricles) dpci. White dashed lines indicate the approximate wound border.

Scale bars: 100 μm, 20 μm in **(D)**.

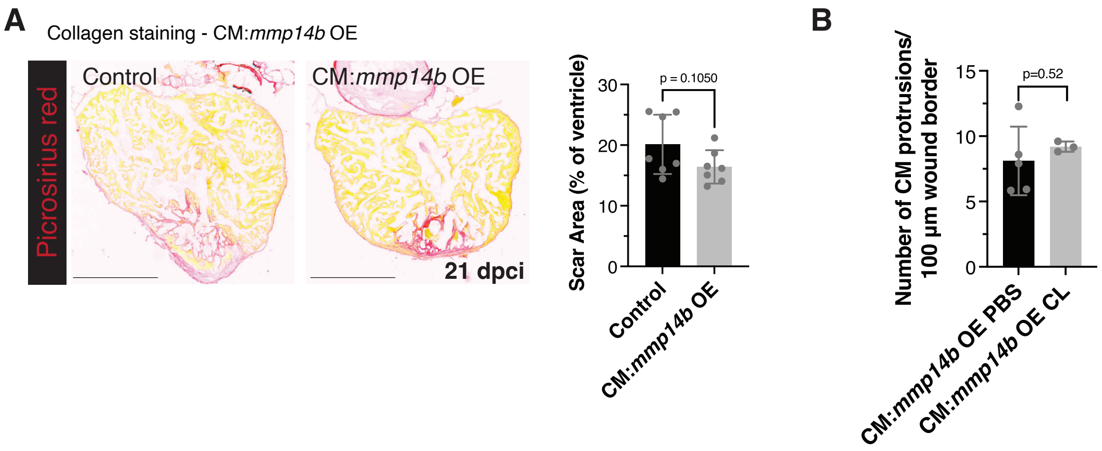

Supplementary Figure 11: *mmp14b* OE in cardiomyocytes does not accelerate heart regeneration.

(A) Picrosirius red staining of collagen in control (n=7 ventricles) and CM:*mmp14b* OE (n=7 ventricles) at 21 dpci (left). Quantification of scar area (% of ventricle area) on the right. Data are presented as mean ±SD. P-value was calculated using an unpaired two-sided t-test. Source data are presented in the Source Data file.

(B) Quantification of the number of CM protrusions per 100 microns of wound border from thick cryosections of ventricles from CM:*mmp14b* OE zebrafish treated with PBS (n=5 ventricles) or clodronate liposomes (n=3 ventricles) at 10 dpci. Data are presented as mean ±SD. P-values were calculated using an unpaired two-sided t-test. Source data are presented in the Source Data file.

Supplementary Table 1: PCR primers used in this study

Primer	Purpose	Sequence (5'-3')	Figure
irf8	HRMA genotyping	ACGGCATACTAGTGAAGTAAAGGT<br>CTATAAGCCACTGTTTCAGTCTGC	
mmp14b WT	genotyping	TGCATTCACACACATACACTGCGAC<br>ATTTACAACCACATCCCCCCTGCC	Supp. 9A
mmp14b deletion	genotyping	AGACATAAGTGAAGAGTGAGAGAGG<br>TGGAGTCTTCGTTAGGGCAG	Supp. 9A
mmp14b ORF	verifying *mmp14b* deletion	CTGTGCATGAGAAGCCGAAGA<br>AGAAGTGCGCCGAGTGTAAA	Fig. 7E
mmp14b WISH	WISH probe	AATTAACCCTCACTAAAGGGAGAATCTGGAGGACACCCTCGAC<br>TAATACGACTCACTATAGGGAGAGGCAGCCCATCCAATCCTTA	Fig. 7B
CM:mmp14b OE	genotyping	aagagcagcctgacaggac<br>CTTGTGGCCGTTTACGTCGC	
rpl13a	qPCR	TCTGGAGGACTGTAAGAGGTATGC<br>AGACGCACAATCTTGAGAGCAG	all RT-qPCR
mmp14b	qPCR	GGAAAATGATCTGGAGCGGGTT<br>AGCCATGCCTCAGGTTTCATA	Supp. 8B, 8D
mmp14b 3'UTR	qPCR	GCGCAGTTACCAAATGCACA<br>CATGGGGTGAGAATGGACCC	Fig. 7F
mmp14a	qPCR	cttcagagtttgggaggccg<br>gtacgcatgggccaggaaac	Fig. 7F<br>Supp. 9B
fn1b	qPCR	GCCATTGGTACTGGATCTGCAG<br>GTTCTGATCCAGCTCATGCCATTG	Supp. 4F<br>Fig. 8E
fn1a	qPCR	AAACTTGGAGCGGCTGCGG<br>CACAGGTGCGATTGAACACG	Supp. 4F<br>Fig. 8E
postnb	qPCR	GACCCGAGTTATCCAGGGAGAG<br>CTCAATCACACGAGTGACCTTGG	Fig. 8E<br>Supp. 10C
fap	qPCR	TTCGCTTGGAGTGGGCGAC<br>TTCATCCCCCTGGAAACCACTTC	Supp. 4F<br>Supp. 10C
vim	qPCR	TCCAGCCGGCAGTACAGCAG<br>CTCAAGCCTTTACTCGCGTAC	Supp. 4F<br>Supp. 10C
col1a1a	qPCR	GCCAGGTCTACAATGACAGGG<br>ATCACTTCGTCGCACATTACGG	Supp. 4F<br>Fig. 8E
col11a1b	qPCR	CCCAAAAGGCCCACCTGGG<br>TGGAAACCAGTCTCTCCTCGC	Supp. 4F<br>Fig. 8E
acta2	qPCR	CTCTGGAGAAGAGCTATGAGCTTC<br>TCCCGATGAAGGACGGCTG	Supp. 4F<br>Supp. 10C
col12a1a	qPCR	TTGACTCTAGTACTCAGTTCAGTAG<br>TTCCTCCATGACATTTGCACTGTG	Supp. 4F<br>Fig. 8E
nppc	qPCR	GGAGCTGTCTGAGTTCCTGG<br>TGTTCGTTCATGATCCGCGC	Supp. 4F<br>Supp. 10C
spock3	qPCR	AGAGATGAGGTGGAGGAACCAG<br>ACATTTTATCTTCAGACATGGATCTTTGG	Supp. 4F<br>Supp. 10C
c4	qPCR	TTCATATGTTCAACTTGCTGCC<br>CCCCGTTTTGTTGACAAATAGATG	Supp. 4F<br>Supp. 10C
cfl1	qPCR	gggactgcatcacagctcattc<br>caatgagccttgcggaccttc	Fig. 6E
pfn1	qPCR	actcactttgcatcggagcag<br>atggactcctgacccggtg	Fig. 6E
pfn2	qPCR	atgtcggacggatcgtgc<br>ttggtgaagaaactctgtcggtct	Fig. 6E
rhoab	qPCR	gccgacattgaagttgacagc<br>gcgtccacttctccggaatattc	Fig. 6E
arpc3	qPCR	ctgcctgctaggtttgggac<br>ggggcagtaatgccatattgcc	Fig. 6E

Primer	Purpose	Sequence (5'-3')	Figure
rac1a	qPCR	gatgcaggccataaagtgtgtg<br>cccacaatcccaggtttactgg	Fig. 6E
tln1	qPCR	cgctgtcgctgaagatcgg<br>cctgcttccagccaaatgcc	Fig. 6E
itga5	qPCR	cccaaagccaacaccagtcag<br>ccactgatgggatttgaactctcc	Fig. 6E
pcolce2b	qPCR	ggaacccctgagagccact<br>gcatacatgctacctcctcggg	Fig. 6E
timp2a	qPCR	tcatcctcaacaggcgttctgc<br>cgcttgatcgggttcccataa	Fig. 6E
