# Supplementary material for: Border-zone cardiomyocytes and macrophages regulate extracellular matrix remodeling to promote cardiomyocyte protrusion during cardiac regeneration
Source: Nat Commun. 2025 Apr 23;16:3823. doi: 10.1038/s41467-025-59169-4 (PMC12019606; doi:10.1038/s41467-025-59169-4)
Supplement: Supplementary file 3 — Description of Additional Supplementary Files [file 41467_2025_59169_MOESM3_ESM.pdf]

**Description of Additional Supplementary Files**

File Name: Supplementary Movie 1
Description: **Timelapse imaging of corticallylocated (Tg(myl7:LIFEACTGFP)+) CM protrusion into the injured area from ventricular sections at 10 dpci.** White line denotes apical wound surface and yellow arrowhead follows the end of a cortical CM protrusion. IA, injured area.

File Name: Supplementary Movie 2
Description: **Tracking of cortically-located CM protrusions from ventricular sections at 10 dpci using TrackMate.** Yellow text denotes the start and end of a CM protrusion moving in a net (+) direction, blue text denotes the start and end of a CM protrusion moving in a net (-) direction; IA, injured area.

File Name: Supplementary Movie 3
Description: **Timelapse imaging of trabecular (Tg(myl7:actn3b-EGFP)+) CM protrusion into the injured area from ventricular sections at 10 dpci.** Yellow arrowhead follows the end of a trabecular CM protrusion into the injured area

File Name: Supplementary Movie 4
Description: **Timelapse imaging of trabecular (Tg(myl7:actn3b-EGFP)+) CM protrusion into the injured area from ventricular sections at 3 dpci.**

File Name: Supplementary Movie 5
Description: **Timelapse imaging of CMs and macrophages in Tg(mpeg1:EGFP); Tg(myl7:mKATE-CAAX) ventricular sections at 10 dpci.**

File Name: Supplementary Movie 6
Description: **Timelapse imaging of border zone Tg(mpeg1:EGFP)+ cells from ventricular sections at 10 dpci.**

File Name: Supplementary Movie 7
Description: **Timelapse imaging of Tg(mpeg1:EGFP)+ cells in the injury from ventricular sections at 10 dpci.**
