# Supplementary material for: Border-zone cardiomyocytes and macrophages regulate extracellular matrix remodeling to promote cardiomyocyte protrusion during cardiac regeneration
Source: Nat Commun. 2025 Apr 23;16:3823. doi: 10.1038/s41467-025-59169-4 (PMC12019606; doi:10.1038/s41467-025-59169-4)
Supplement: Supplementary file 11 — Reporting Summary [file 41467_2025_59169_MOESM11_ESM.pdf]

nature portfolio

Corresponding author(s): Arica Beisaw
Last updated by author(s): Mar 28, 2025

Reporting Summary

Nature Portfolio wishes to improve the reproducibility of the work that we publish. This form provides structure for consistency and transparency in reporting. For further information on Nature Portfolio policies, see our Editorial Policies and the Editorial Policy Checklist.

Statistics

For all statistical analyses, confirm that the following items are present in the figure legend, table legend, main text, or Methods section.

n/a	Confirmed	
☐	☒	The exact sample size (*n*) for each experimental group/condition, given as a discrete number and unit of measurement
☐	☒	A statement on whether measurements were taken from distinct samples or whether the same sample was measured repeatedly
☐	☒	The statistical test(s) used AND whether they are one- or two-sided<br>*Only common tests should be described solely by name; describe more complex techniques in the Methods section.*
☒	☐	A description of all covariates tested
☐	☒	A description of any assumptions or corrections, such as tests of normality and adjustment for multiple comparisons
☐	☒	A full description of the statistical parameters including central tendency (e.g. means) or other basic estimates (e.g. regression coefficient) AND variation (e.g. standard deviation) or associated estimates of uncertainty (e.g. confidence intervals)
☐	☒	For null hypothesis testing, the test statistic (e.g. *F*, *t*, *r*) with confidence intervals, effect sizes, degrees of freedom and *P* value noted<br>*Give P values as exact values whenever suitable.*
☒	☐	For Bayesian analysis, information on the choice of priors and Markov chain Monte Carlo settings
☒	☐	For hierarchical and complex designs, identification of the appropriate level for tests and full reporting of outcomes
☒	☐	Estimates of effect sizes (e.g. Cohen's *d*, Pearson's *r*), indicating how they were calculated

Our web collection on statistics for biologists contains articles on many of the points above.

Software and code

Policy information about availability of computer code

Data collection	Nikon NIS-Elements (AR-version) software was used in the acquisition of time-lapse confocal imaging data (Nikon AX). Leica LAS X software was used in the acquisition of confocal imaging and histological staining (bright-field) data (Leica Mica). ZEN software from Zeiss was used to acquire confocal imaging data (Zeiss LSM 800).
Data analysis	ImageJ open source imaging processing software (v2.9.0) was used to process and quantify all confocal imaging data. For time-lapse imaging, ImageJ plug-in StackReg (v2.0.0) was used to correct for drift from live-imaging data and the TrackMate plug-in (v7.10.2) was used to manually track CM protrusion ends. For single-cell RNA-sequencing data, Scanpy (v1.9) was used for analysis, visualization, and differential gene expression analysis. Panther (v18.0) was used for gene ontology enrichment analysis and REVIGO (v1.8.1) was used for visualization of gene ontology enrichment. Integrated Genome Viewer (IGV, v2.8.9) was used for visualizing ATAC-sequencing data tracks. GraphPad Prism 10 was used for data visualization and statistical analysis.

For manuscripts utilizing custom algorithms or software that are central to the research but not yet described in published literature, software must be made available to editors and reviewers. We strongly encourage code deposition in a community repository (e.g. GitHub). See the Nature Portfolio guidelines for submitting code & software for further information.

April 2023

Data

Policy information about availability of data

All manuscripts must include a data availability statement. This statement should provide the following information, where applicable:

- Accession codes, unique identifiers, or web links for publicly available datasets
- A description of any restrictions on data availability
- For clinical datasets or third party data, please ensure that the statement adheres to our policy

Single-cell RNA-seq data from this study have been deposited in the Gene Expression Omnibus (GEO) database under the accession number: GSE251856

Research involving human participants, their data, or biological material

Policy information about studies with human participants or human data. See also policy information about sex, gender (identity/presentation), and sexual orientation and race, ethnicity and racism.

Reporting on sex and gender	*Use the terms sex (biological attribute) and gender (shaped by social and cultural circumstances) carefully in order to avoid confusing both terms. Indicate if findings apply to only one sex or gender; describe whether sex and gender were considered in study design; whether sex and/or gender was determined based on self-reporting or assigned and methods used. Provide in the source data disaggregated sex and gender data, where this information has been collected, and if consent has been obtained for sharing of individual-level data; provide overall numbers in this Reporting Summary. Please state if this information has not been collected. Report sex- and gender-based analyses where performed, justify reasons for lack of sex- and gender-based analysis.*
Reporting on race, ethnicity, or other socially relevant groupings	*Please specify the socially constructed or socially relevant categorization variable(s) used in your manuscript and explain why they were used. Please note that such variables should not be used as proxies for other socially constructed/relevant variables (for example, race or ethnicity should not be used as a proxy for socioeconomic status). Provide clear definitions of the relevant terms used, how they were provided (by the participants/respondents, the researchers, or third parties), and the method(s) used to classify people into the different categories (e.g. self-report, census or administrative data, social media data, etc.) Please provide details about how you controlled for confounding variables in your analyses.*
Population characteristics	*Describe the covariate-relevant population characteristics of the human research participants (e.g. age, genotypic information, past and current diagnosis and treatment categories). If you filled out the behavioural & social sciences study design questions and have nothing to add here, write "See above."*
Recruitment	*Describe how participants were recruited. Outline any potential self-selection bias or other biases that may be present and how these are likely to impact results.*
Ethics oversight	*Identify the organization(s) that approved the study protocol.*

Note that full information on the approval of the study protocol must also be provided in the manuscript.

Field-specific reporting

Please select the one below that is the best fit for your research. If you are not sure, read the appropriate sections before making your selection.

☒ Life sciences ☐ Behavioural & social sciences ☐ Ecological, evolutionary & environmental sciences

For a reference copy of the document with all sections, see nature.com/documents/nr-reporting-summary-flat.pdf

Life sciences study design

All studies must disclose on these points even when the disclosure is negative.

Sample size	Sample sizes were determined based on similar studies in our field.
Data exclusions	One irf8 wild-type ventricle from RT-qPCR analysis of fibroblast markers at 10 dpci was excluded, as it was an outlier in all genes measured. For immunostaining and histological analyses, cryoinjured ventricles were excluded if the injury was superficial and there was no significant presence of trabecular cardiomyocytes at the wound border.
Replication	All time-lapse imaging, immunostaining, histological staining, and RT-qPCR analyses were performed in at least 3 independent biological replicates. All attempts at replication were successful. Previously published ATAC-seq data was performed in sorted cells from 2 independent biological replicates. RT-qPCR analysis from sorted cardiomyocytes (for mmp14b expression analysis) was performed in 2 independent biological replicates.
Randomization	Organisms for all phenotypic characterizations were randomly chosen among each genotype population.
Blinding	Blinding to group allocation was not performed prior to analysis. For confocal image/immunostaining/histological staining analysis,

investigators were blinded during quantification of number of CM protrusions, length of CM protrusions, mpeg1+ cell number, scar area, and CHP/Aldh1a2 intensity between control and mutant groups to reduce bias.

Reporting for specific materials, systems and methods

We require information from authors about some types of materials, experimental systems and methods used in many studies. Here, indicate whether each material, system or method listed is relevant to your study. If you are not sure if a list item applies to your research, read the appropriate section before selecting a response.

Materials & experimental systems

n/a	Involved in the study
☐	☒ Antibodies
☒	☐ Eukaryotic cell lines
☒	☐ Palaeontology and archaeology
☐	☒ Animals and other organisms
☒	☐ Clinical data
☒	☐ Dual use research of concern
☒	☐ Plants

Methods

n/a	Involved in the study
☒	☐ ChIP-seq
☒	☐ Flow cytometry
☒	☐ MRI-based neuroimaging

Antibodies

Antibodies used

1. Mouse anti-PCNA, Abcam, ab29, clone PC10, 1:200
2. Rabbit anti-Mef2, Boster, DZ01398-1, 1:200
3. Chicken anti-GFP, Aves Lab, GFP-1020, 1:500
4. Living colors rabbit anti-DsRed, Takara, 632496, 1:300
5. Rat anti-mCherry, Thermo Fisher, M11217, clone 16D7, 1:200
6. Mouse anti-MYH1, DSHB, A4.1025, 1:100
7. Rabbit anti-Col12a1a, Boster, DZ41260, 1:200
8. Rabbit anti-Aldh1a2, Genetex, GTX124302, 1:100
9. Collagen Hybridizing Peptide, 3Helix, B-CHP BIO60, 20uM
10. Mouse anti-RFP, Invitrogen, MA5-15257, 1:500
11. Mouse anti-Tnfa, Abcam, ab1793, 1:100
12. Rabbit anti-Cxcr4b, Abcam, ab229623, 1:500
13.Mouse anti-Vcl, Sigma-Aldrich, V9131, 1:250
14. Mouse anti-embCMHC, DSHB, N2.261, 1:25

Validation

1. https://www.abcam.com/products/primary-antibodies/pcna-antibody-pc10-ab29.html
2. https://www.bosterbio.com/polyclonal-anti-mef2-antibody-dz01398-1-boster.html
3. https://www.aveslabs.com/products/anti-green-fluorescent-protein-antibody-gfp
4. https://www.takarabio.com/documents/Certificate%20of%20Analysis/632496/632496-101717.pdf
5. https://www.thermofisher.com/antibody/product/mCherry-Antibody-clone-16D7-Monoclonal/M11217
6. https://dshb.biology.uiowa.edu/A4-1025
7. https://www.bosterbio.com/anti-zebrafish-col12a1a-antibody-dz41260-boster.html
8. https://www.genetex.com/Product/Detail/Aldh1a2-antibody/GTX124302
9. https://www.3helix.com/products/collagen-hybridizing-peptide-biotin-conjugate-b-chp
10. https://www.thermofisher.com/antibody/product/RFP-Antibody-clone-RF5R-Monoclonal/MA5-15257
11. https://www.abcam.com/en-us/products/primary-antibodies/tnf-alpha-antibody-52b83-ab1793?srsltid=AfmBOoqPzUt4scKw609sO-M4gX6dIkHjrQNuSkhwnnMNehmEdyp_ORJL
12. https://www.abcam.com/en-us/products/primary-antibodies/cxcr4b-antibody-n-terminal-ab229623?srsltid=AfmBOooq1WqkoNK17nA6c6d0wCa9dv0lbtUcotA3Tp74AOm6m_FK-zpn
13. https://www.sigmaaldrich.com/DE/de/product/sigma/v9131?srsltid=AfmBOoqkTwzs-gcJyUiuzHvfZunQWexAjNYxizxwF_DlY0m8azK8cvwj
14. https://dshb.biology.uiowa.edu/N2-261

Animals and other research organisms

Policy information about studies involving animals; ARRIVE guidelines recommended for reporting animal research, and Sex and Gender in Research

Laboratory animals: Zebrafish (AB strain) from ages 4-12 months were used in this study.

Wild animals: This study did not involve wild animals.

Reporting on sex	Both male and female zebrafish were used in this study and no sex-based analysis was performed.
Field-collected samples	This study did not include samples collected from the field.
Ethics oversight	All zebrafish husbandry and experimentation were performed under standard conditions in accordance with institutional (MPG and Heidelberg University) and national (RP Darmstadt and RP Karlsruhe) ethical and animal welfare guidelines.

Note that full information on the approval of the study protocol must also be provided in the manuscript.

Plants

Seed stocks	*Report on the source of all seed stocks or other plant material used. If applicable, state the seed stock centre and catalogue number. If plant specimens were collected from the field, describe the collection location, date and sampling procedures.*
Novel plant genotypes	*Describe the methods by which all novel plant genotypes were produced. This includes those generated by transgenic approaches, gene editing, chemical/radiation-based mutagenesis and hybridization. For transgenic lines, describe the transformation method, the number of independent lines analyzed and the generation upon which experiments were performed. For gene-edited lines, describe the editor used, the endogenous sequence targeted for editing, the targeting guide RNA sequence (if applicable) and how the editor was applied.*
Authentication	*Describe any authentication procedures for each seed stock used or novel genotype generated. Describe any experiments used to assess the effect of a mutation and, where applicable, how potential secondary effects (e.g. second site T-DNA insertions, mosiacism, off-target gene editing) were examined.*
