# Supplementary material for: Border-zone cardiomyocytes and macrophages regulate extracellular matrix remodeling to promote cardiomyocyte protrusion during cardiac regeneration
Source: Nat Commun. 2025 Apr 23;16:3823. doi: 10.1038/s41467-025-59169-4 (PMC12019606; doi:10.1038/s41467-025-59169-4)
Supplement: Supplementary file 2 — Peer Review file [file 41467_2025_59169_MOESM2_ESM.pdf]

nature portfolio

Peer Review File

Border-zone cardiomyocytes and macrophages regulate extracellular matrix remodeling to promote cardiomyocyte protrusion during cardiac regeneration

Corresponding Author: Dr Arica Beisaw

This manuscript has been previously reviewed at another journal. This document only contains information relating to versions considered at Nature Communications.

This file contains all reviewer reports in order by version, followed by all author rebuttals in order by version.

Version 1:

Reviewer comments:

Reviewer #1

(Remarks to the Author)
All of my comments from the previous submission have been adequately addressed by the authors.

Reviewer #3

(Remarks to the Author)
The authors have addressed all my previous concerns and significantly improved the manuscript. I have no further comments.

Open Access This Peer Review File is licensed under a Creative Commons Attribution 4.0 International License, which

permits use, sharing, adaptation, distribution and reproduction in any medium or format, as long as you give appropriate credit to the original author(s) and the source, provide a link to the Creative Commons license, and indicate if changes were made.
In cases where reviewers are anonymous, credit should be given to 'Anonymous Referee' and the source.
The images or other third party material in this Peer Review File are included in the article’s Creative Commons license, unless indicated otherwise in a credit line to the material. If material is not included in the article’s Creative Commons license and your intended use is not permitted by statutory regulation or exceeds the permitted use, you will need to obtain permission directly from the copyright holder.
To view a copy of this license, visit https://creativecommons.org/licenses/by/4.0/
